# Heroin-Related Fatalities in Jeddah, Saudi Arabia, between 2008 and 2018

**DOI:** 10.3390/toxics11030248

**Published:** 2023-03-06

**Authors:** Ahmed I. Al-Asmari, Hassan Alharbi, Abdulnasser E. Al-Zahrani, Torki A. Zughaibi

**Affiliations:** 1 Laboratory Department, Ministry of Health, King Abdul-Aziz Hospital, Jeddah 21442, Saudi Arabia; 2 King Fahd Medical Research Center, King Abdulaziz University, Jeddah 21589, Saudi Arabia; 3 Poison Control and Forensic Chemistry Center, Ministry of Health, Jeddah 21176, Saudi Arabia; 4 Department of Medical Laboratory Sciences, Faculty of Applied Medical Sciences, King Abdulaziz University, Jeddah 21589, Saudi Arabia

**Keywords:** forensic toxicology, opiates, opioids, LC-MS/MS, postmortem

## Abstract

To date, epidemiological studies have not evaluated heroin-related deaths in the Middle East and North African regions, especially Saudi Arabia. All heroin-related postmortem cases reported at the Jeddah Poison Control Center (JPCC) over a 10-year period (21 January 2008 to 31 July 2018) were reviewed. In addition, liquid chromatography electrospray ionization tandem mass spectrometry (LC/ESI-MS/MS) was utilized to determine the 6-monoacetylmorphine (6-MAM), 6-acetylcodeine (6-AC), morphine (MOR), and codeine contents in unhydrolyzed postmortem specimens. Ninety-seven heroin-related deaths were assessed in this study, and they represented 2% of the total postmortem cases at the JPCC (median age, 38; 98% male). In the blood, urine, vitreous humor, and bile samples, the median morphine concentrations were 280 ng/mL, 1400 ng/mL, 90 ng/mL, and 2200 ng/mL, respectively; 6-MAM was detected in 60%, 100%, 99%, and 59% of the samples, respectively; and 6-AC was detected in 24%, 68%, 50%, and 30% of the samples, respectively. The highest number of deaths (33% of total cases) was observed in the 21–30 age group. In addition, 61% of cases were classified as “rapid deaths,” while 24% were classified as “delayed deaths.” The majority (76%) of deaths were accidental; 7% were from suicide; 5% were from homicide; and 11% were undetermined. This is the first epidemiological study to investigate heroin-related fatalities in Saudi Arabia and the Middle East and North African region. The rate of heroin-related deaths in Jeddah remained stable but increased slightly at the end of the study period. Most patients were heroin-dependent abusers and from the middle-aged group. The availability of urine, vitreous humor, and bile specimens provided valuable information regarding the opioids that were administered and the survival time following heroin injection.

## 1. Introduction

The abuse of heroin (diamorphine, acetomorphine, and diacetylmorphine) remains a major cause of death worldwide. For example, an estimated 62 million people used opioids for non-medical purposes in 2019 (1.2% of the global population), with half using heroin or opium [1]. The primary cause of heroin-related deaths is overdose [2,3,4], while indirect causes of death include infectious diseases, such as human immunodeficiency virus (HIV) and hepatitis, and sepsis associated with intravenous injection [2]. The latest United Nations Office on Drug and Crime (UNDOC) report indicated that in 2019, heroin trafficking occurred in ninety-nine countries [1]. According to a recent report from the European Union, heroin is the most abused opioid. In 2019, 85% of the drug-related deaths in Europe were caused by one or more opioids [5]. Currently, North America is experiencing a sharp rise in heroin users, with an increase of approximately 150% from 2007 to 2017 [1]. In the United States, Evans et al. analyzed drug residues from needle-exchange syringes and found that heroin was the second most detected controlled substance [6]. In the last UNODC report published in 2022 [1], it was stated that although heroin is not the primary cause of death related to opioids in the USA, it is still causing fatalities via ingestion, either intentionally or accidentally. It has been estimated that 9.5 million people were using opioids non-medically in the past year in the USA. Almost 98% of them were using pharmaceutical opioids non-medically; in this period, 9.5% were using heroin, and 7.4% used both heroin and pharmaceutical opioids. Based on another report [7], heroin alone as a cause of death seems to be declining; however, heroin-related fatalities have been increasing in terms of deaths in that region. Heroin found in such cases was allegedly ingested accidentally, as most cases were also fentanyl-related fatalities, and this is most likely due to the mixing of heroin with fentanyl in the black market. According to a UNODC report, heroin is still the predominant opioid used in many Asian countries, such as India and Pakistan. In 2018, 23 million Indians were estimated to use opioids, and heroin was the most frequently used drug among the population. In contrast, a 50% decline in heroin use has been reported among registered drug users in China [1].

In the Middle East and North African (MENA) region, principally in the Arabian Gulf region, heroin is reported in the media as a symbol of drug addiction. However, heroin-related fatalities have rarely been reported in scientific literature from this region. Thus, little is known about heroin-related deaths, and most studies in the Arabian Gulf region are outdated [8,9]. Al-Matrouk et al. reviewed these studies and found that they were mostly survey-based and lacked laboratory-based research; thus, these studies did not actually describe the real situation of drug abuse patterns in these countries [10].

In Saudi Arabia, alcohol and drug use are prohibited by law and denounced based on social and religious perspectives [11,12]. Thus, Saudi drug use is poorly understood. In one study, the abuse of heroin in recent years appeared to decrease or was not reported in certain regions of Saudi Arabia, such as the Al-Qassim region [13]. In contrast, heroin was the second most detected drug at Jeddah’s Addiction Hospital [8]. In the eastern region of Saudi Arabia, 49% of overdose deaths were related to opiates (116 out of 249 drug-related deaths) in an 8-year period from 1990 to 1997 [14]. However, in that study, postmortem details were not reported; thus, whether heroin or codeine was the main opioid was not determined.

Heroin is unstable in biological specimens and rarely identified in postmortem cases [15]. Alternatively, its biomarkers (6-monoacetylmorphine (6-MAM) and 6-acetylcodeine (6-AC)) provide sufficient evidence to demonstrate whether heroin was the opioid administered and whether death occurred shortly following ingestion. 6-MAM has a short half-life of ~40 min before converting to morphine [16]. However, 6-AC metabolism and its role in heroin-related fatalities have rarely been reported [17,18]. In cases of death, if sample collection is delayed, then heroin biomarkers will be converted to morphine and codeine. Morphine is an active metabolite of heroin with a much longer half-life than heroin and 6-MAM, whereas codeine is formed by 6-AC degradation [2]. In fact, 6-MAM, morphine, and morphine metabolites have frequently been reported in heroin-related deaths, whereas the presence of 6-AC in postmortem cases has rarely been reported [19,20], which may be due to its short half-life, instability under storage conditions, and incredibly low concentrations in deceased specimens [15,20]. To the best of our knowledge, no epidemiological studies have reported 6-MAM and 6-AC together in a postmortem specimen in heroin-related fatalities.

The patterns of heroin-related deaths are poorly understood in the MENA region. Thus, up-to-date epidemiological studies to reveal heroin-related fatalities and explore the problem of heroin abuse are a crucial task. As Jeddah is one of the major cities in Saudi Arabia (4 million population), this paper reports the first epidemiological study evaluating heroin-related deaths in Jeddah City, Saudi Arabia, between 2008 and 2018 and investigates whether metabolites of heroin found in various bodily fluid samples can be used to identify the type of opioid ingested and the time of death after administration. Such toxicological information is crucial for providing knowledge on the trends in illegal drug use and planning initiatives to reduce accidents and deaths among drug users.

## 2. Materials and Methods

### 2.1. Reagents and Standards

Morphine, morphine-d3, 6-MAM, 6-monoacetylmorphine-d3 (6-MAM-d3), codeine, codeine-d3, and 6-AC were purchased from Lipomed (Arlesheim, Switzerland). Methanol (HPLC grade), acetonitrile (HPLC grade), ammonium carbonate, formic acid, and ammonium hydroxide were obtained from BDH (Poole, UK). Ammonium formate was obtained from Sigma-Aldrich (Steinheim, Germany). Clean Screen^®^ solid phase extraction (SPE) cartridges (CSDAU203) were obtained from United Chemical Technologies (Bristol, PA, USA).

### 2.2. Solid Phase Extraction (SPE)

One milliliter of each specimen was placed in a glass test tube, which was then spiked with 50 µL of the internal standard (containing 50 µg/mL of 6-MAM-d3, morphine-d3, and codeine-d3). The mixture was then mixed and vortexed for at least 10 s. Next, 2 mL of 0.1 M phosphate buffer (pH 6) was added to the samples, mixed, and centrifuged for 10 min at 3500 rpm. Before loading the samples into the SPE cartridges, the samples were prepared for SPE by adding 2 mL of methanol, 2 mL of deionized water (D.H_2_O), and 2 mL of 0.1 M phosphate buffer adjusted to pH 6. The sample mixture was then loaded onto the SPE column using gravity. Next, the cartridges were washed by adding 1 mL of D.H_2_O, followed by 1 mL of acetic acid (0.1 M), and then dried under vacuum for 5 min. The third washing step was completed by adding 2 mL of hexane. Two elution steps were performed: the first elution (A) was performed by adding 2 mL hexane/ethyl acetate (1:1, *v*/*v*). Next, the elution tubes were removed, and the SPE cartridges were washed using 3 mL of methanol and then dried under full vacuum for 2 min. The second elution (B) was performed by adding 3 mL of dichloromethane/isopropanol/ammonium hydroxide (78:20:2, *v*/*v*) to each cartridge. Both fractions were collected in a glass evaporation tube and evaporated to dryness using nitrogen. Finally, 200 μL of the initial mobile phase was added to the final extracts and subjected to liquid chromatography tandem mass spectrometry (LC-MS/MS) using an injection volume of 1.0 μL.

### 2.3. LC-MS/MS Systems

Two different LC-MS/MS systems were used to analyze the heroin biomarkers, morphine, and codeine. The first system included a Thermo Finnigan LCQ Fleet ion trap instrument equipped with a Surveyor LC system interface (Thermo Finnigan, San Jose, USA) equipped with electrospray ionization (ESI+) and selective reaction monitoring modes. Analytes were separated using a Synergy Polar RP column (150 × 2.0 mm, 4 μm particle size, Phenomenex, Torrance, CA, USA) equipped with a guard column with identical packing material (4 × 2.0 mm, Phenomenex, Torrance, CA, USA). The column oven and auto-sampler tray temperatures were kept at 30 °C and 4 °C, respectively. The gradient mobile phase consisted of ammonium formate buffer (10 mM, pH 3) as mobile phase A and an organic modifier of 100% acetonitrile as mobile phase B, with a flow rate of 0.3 mL/min for the whole run. The gradient program was initiated by applying 3% B for 3 min, which was increased to 15% over the next 5 min, 26% over the next 7 min, 80% over the next 13 min, and 95% over the next two min. After 27 min, the initial mobile phase was applied for 3 min. Data were acquired and managed using the Xcalibur system (Version 2.07 SP1, Thermo Finnigan, San Jose, USA).

The second LC-MS/MS analysis was performed according to a previously reported method [20]. Brifly, LC-MS/MS with a triple quadrupole mass spectrometer (Shimadzu LCMS-8050, Kyoto, Japan), (+)ESI, and a Shimadzu Nexera UHPLC system were used for the analysis of 6-MAM, 6-AC, morphine, and codeine. Analytes of interest were separated using a phenyl LC-column (Raptor Biophenyl column (50 × 3.0 mm, 2.7 μm, Restek, USA) fitted with a guard column with a similar chemistry (Raptor Biophenyl column (5.0 × 3.0 mm, 2.7 µm, Restek, USA). The gradient condition mobile phase consisted of ammonium formate (10 mM, pH 3, mobile phase A) and 100% methanol (mobile phase B), and the flow rate was 0.3 mL/min for the whole run. The mobile phase gradient elution started with 3% B during the first minute and then increased to 5% within 1 min and 95% over the next 13 min. The initial mobile phase was then applied for 1 min and maintained for the next 4 min to re-equilibrate and prepare the column for the next injection. The LC-MS/MS parameters are listed in Table S1. Data were acquired and managed using LabSolution software (version 5.75, Shimadzu, Kyoto, Japan). The LC-MS/MS parameters are listed in Table 1.

### 2.4. Case Samples

#### 2.4.1. Ethical Approval

This study was approved by the IRB committee of Jeddah Health Affairs, Ministry of Health, Jeddah, Saudi Arabia (research no: #A00221; approval no: A00187).

#### 2.4.2. Sample Collection

Blood samples were collected from the subclavian site in tubes containing 1% sodium fluoride (BNaF). Vitreous humor fluid was collected in gray tubes containing sodium fluoride, and urine and bile samples were stored in a plain container without any preservative. All samples were stored frozen (at −20 °C) until analysis. Autopsy samples were thawed to obtain them and refrozen until use. Blood samples were obtained for 84 postmortem cases (87%), urine samples were obtained for 74 cases (76%), vitreous humor samples were obtained from two eyes in 70 cases (72%), and bile specimens were obtained for 27 cases (28%).

#### 2.4.3. Data Collecting for Post-Mortem Cases

All postmortem body fluids and tissues collected for forensic toxicology investigations are analyzed for commonly abused drugs and reported, and they are also assessed based on requests by forensic pathologists. All heroin-related postmortem cases reported at the Jeddah Poison Control and Medical Chemistry Center (JPCC) over the last 10-years (21 January 2008 to 31 July 2018) were reviewed. The 6-MAM, 6-AC, morphine, and codeine contents were reviewed, and case details were collected from the online Forensic Toxicology Jeddah Reports Database. The search was conducted between February 2015 and July 2018. Data before February 2015 was manually collected from JPCC archive files. Cases positive for heroin use were reviewed and included in this investigation according to the inclusion/exclusion criteria mentioned below. Ninety-seven cases reported during the study period were included.

#### 2.4.4. Inclusion/Exclusion Criteria

The same criteria applied in our previous study were used in the current study [21]. The most important criteria included the detection of 6-MAM in blood samples available for testing or in any alternative samples, particularly vitreous humor, urine, and bile.

#### 2.4.5. Other Toxicological Investigations

All postmortem cases, irrespective of the manner of death, were screened using a common immunoassay reagent. The second step was to search for other concomitant drugs using general unknown screening approaches (GUS), (known as systemic toxicological analysis, or STA) by gas chromatography (GC) coupled to a flame ionization detector (FID), gas chromatography coupled to mass spectrometry (GC-MS), and LC-MS/MS.

The analysis methods were all fully validated using international guidelines for forensic investigation [22], and a selectivity study was conducted that included additional toxicology testing for drugs and their metabolites that are commonly detected in postmortem forensic toxicology. The analyses used whole blood, urine, or tissue specimens when other body fluid samples were not available. STA includes immunoassay testing, which employs two separate instruments: alcohol testing using GC-Headspace-FID, carbon monoxide testing using spectrometric techniques, heavy metal analysis using inductively coupled plasma mass spectrometry, and GUS using GC-MS and LC-MS/MS to confirm all suspected positive results. GUS depends on the case, and target drugs and their metabolites in specimens of interest, which include but are not limited to opiates, opioids, amphetamines, cocaine, benzodiazepines, barbiturates, antipsychotics, cannabinoids, and their metabolites, were identified using adapted LC-MS/MS methods as previously reported [23,24,25], while GUS for non-target drugs was performed using GC-MS [26,27].

### 2.5. Statistical Analysis

Useful statistical data are reported in terms of the frequency, percentage, mean, median, and range when applicable. The data were calculated using Statistical Packages for Software Sciences (SPSS) version 28.0.1.1 (Armonk, New York, IBM Corporation) and Microsoft Excel version 16.66.1 (Microsoft, Redmond, WA, USA). Descriptive statistics were completed, and continuous data were presented as median, minimum, and maximum. Definite data were displayed as frequency and percentage. A Mann-Whitney U test was employed to estimate variations between groups. Spearman’s correlation test (R) was utilized to compare variables. A *p*-value <0.05 was considered statistically significant.

### 2.6. Method Validation

The method in the current investigation was validated according to ANSI/ASB standards [22] and other published method validation protocols [28,29]. Two different LC-MS/MS methods were fully validated using the BNaF, urine, vitreous humor, and bile samples before being employed in the current investigative analysis. Complete method validation using blood and other specimens has been published previously [20,25]. Negative human postmortem specimens that were confirmed to be drug-free were utilized for calibration and quality control. Over the 10-year study period, the method parameters were re-optimized and re-validated when needed as part of the quality control and policy and procedure protocol updates, as required by the JPCC. Calibration curves of the matrices of interest were prepared for each new batch of samples according to the sample type (each calibrator was run in duplicate). Linear dynamic range (LDR) was chosen as the quantitative analysis method according to previous reports [20], using a 10-point calibration curve (1000, 500, 250, 100, 50, 25, 10, 5, 1, and 0.5 ng/mL). For each matrix of interest, three positive quality control (QC) standards were established at low, medium, and high analyte concentrations (25 ng/mL, 100 ng/mL, and 800 ng/mL, respectively), which were analyzed on the same day, and this was repeated on five consecutive days (five replicates for each concentration) to investigate the within-run and between-run precision.

Heroin biomarkers are not stable, which leads to a decrease in the concentration of these biomarkers in tested specimens that are not stored properly; therefore, a sensitive method is required to measure these biomarkers. The stability of these heroin biomarkers is a well-known issue that must be considered during sample processing, namely, during sample storage, preparation, and analysis in an autosampler. Therefore, all specimens were immediately frozen, thawed before extraction, and then immediately refrozen after sampling. The stability of these heroin biomarkers was investigated in an autosampler using three controls that were previously used in precision studies, and they were reanalyzed after 24 h, 48 h, and one week.

The most important feature of such analysis methods is to investigate the limit of detection (LOD) in the proposed matrix of interest and the workable lower limit of quantification (LOQ) that can be utilized to quantify these unstable analytes at very low concentrations (≤1 ng/mL). Elevated morphine concentrations in urine and bile were used as the upper limit of quantification (ULOQ) and assessed for all heroin-related analytes. The sensitivity analysis was performed in accordance with the ANSI/ASB method validation guidelines [22].

In each batch of samples, negative blank samples from different matrices were included without any standards or internal standards. Negative blank samples with internal standards were only used to investigate method selectivity, and negative blank samples were run following the calibrator with the highest concentration to investigate any carryover. Matrix effects were investigated using post-extraction addition as reported by Matuszewski et al. [28]. Six different autopsy specimens were analyzed for each matrix of interest using the optimized method. These matrices were negative, and three concentrations, such as those used for precision and accuracy studies, were analyzed (each concentration was repeated five times). The same approach was used to calculate extraction recoveries for these different matrix sources, while standards were added before extraction and internal standards were added post-extraction for the recovery experiment. Matrix effects were obtained by comparison with neat standards prepared in the initial mobile phase, and the extraction recovery value was assessed via comparison with the matrix effect results as described by Matuszewski et al. [28].

## 3. Results

### 3.1. Method Validation

The validated analysis method was acceptable (Table S1), and the sensitivity of the proposed method for detecting incredibly low concentrations of heroin biomarkers and higher concentrations of morphine in urine and bile had an LOQ of 1 ng/mL for all analytes. As indicated in Table S1, the autosampler was set to 4 °C during the stability experiments, which showed that the three controls were stable up to one week, with concentrations of analytes of interest expressed as percentages to their target concentration, which were within ±10%. As most heroin users are known to be polydrug users, the ability of the method to distinguish between analytes of interest and other co-ingested drugs is crucial, especially when opioids with similar drug chemistry can be used. Peaks were not observed in the chromatogram following the injection of blank samples alone, internal standards, or standards of the analytes of interest. Similar results were obtained when only internal standards were injected and no response to heroin biomarkers, morphine, or codeine was observed. This confirmed that both the standards and the internal standards were pure.

Linearity was accepted, with coefficients of determination greater than 0.99 for 6-MAM, 6-AC, morphine, and codeine in multiple body fluid samples. The LOD values were estimated from ten different calibration curves and ranged between 0.2 and 0.4 ng/g, while the LOQ was evaluated by spiking with 1 ng/mL of each analyte of interest, and the results were calculated using freshly prepared calibration curves for all analytes of interest in different matrices of interest. The precision, accuracy, dilution, and autosampler stability results were all within 15% of the nominal value, thus confirming that the method is suitable for the quantification of target analytes in the matrices of interest. In addition, carryover contamination from the previous positive control test was not detected.

### 3.2. Case Samples

#### 3.2.1. Demographic Profile

**Figure 1:**
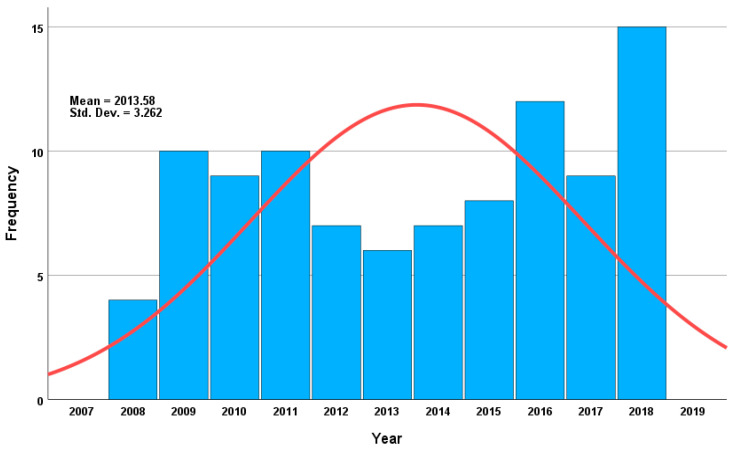
The total number of heroin-related deaths cases over the 10-year period in Jeddah, Saudi Arabia (2008–2018).

**Figure 2:**
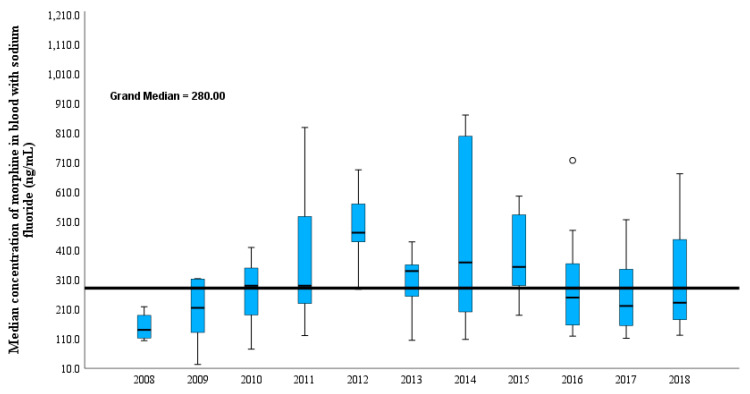
Variation in median concentration of morphine in blood with sodium fluoride in heroin-related deaths cases over a 10-year period. The horizontal boxes correspond to the median concentration ratio, and the box lengths correspond to the 25–75th percentile. The whiskers correspond to the smallest and largest value within 1.5 times the interquartile range, and circles (outlier) symbolize values exceeding at least 1.5 times the interquartile range and extremes (asterisks) correspond to values exceeding at least 3.0 times the interquartile range.

The number of heroin-related deaths in the city of Jeddah within the study period represents 2% of the total number of postmortem cases received by the JPCC between 2008 and 2018 (Figure 1). Although the Jeddah population has increased in recent years, the number of heroin-related fatalities has remained unchanged, with a median of 9 cases per year (ranging 4–15 cases/year). Almost 70% of the heroin-related deaths occurred among Saudi citizens, while 30% occurred among other nationalities. In addition, 64% of the deceased were unemployed and supported by their families. As indicated in Figure 2 and Table S2, the median BNaF morphine concentration was 282 ng/mL (*n* = 85; range, 23–4400 ng/mL); the highest median BNaF morphine concentration occurred in 2012 (470 ng/mL), while the lowest median concentration occurred in 2008 (141 ng/mL).

#### 3.2.2. Age Groups and Analyte Concentrations

**Figure 3:**
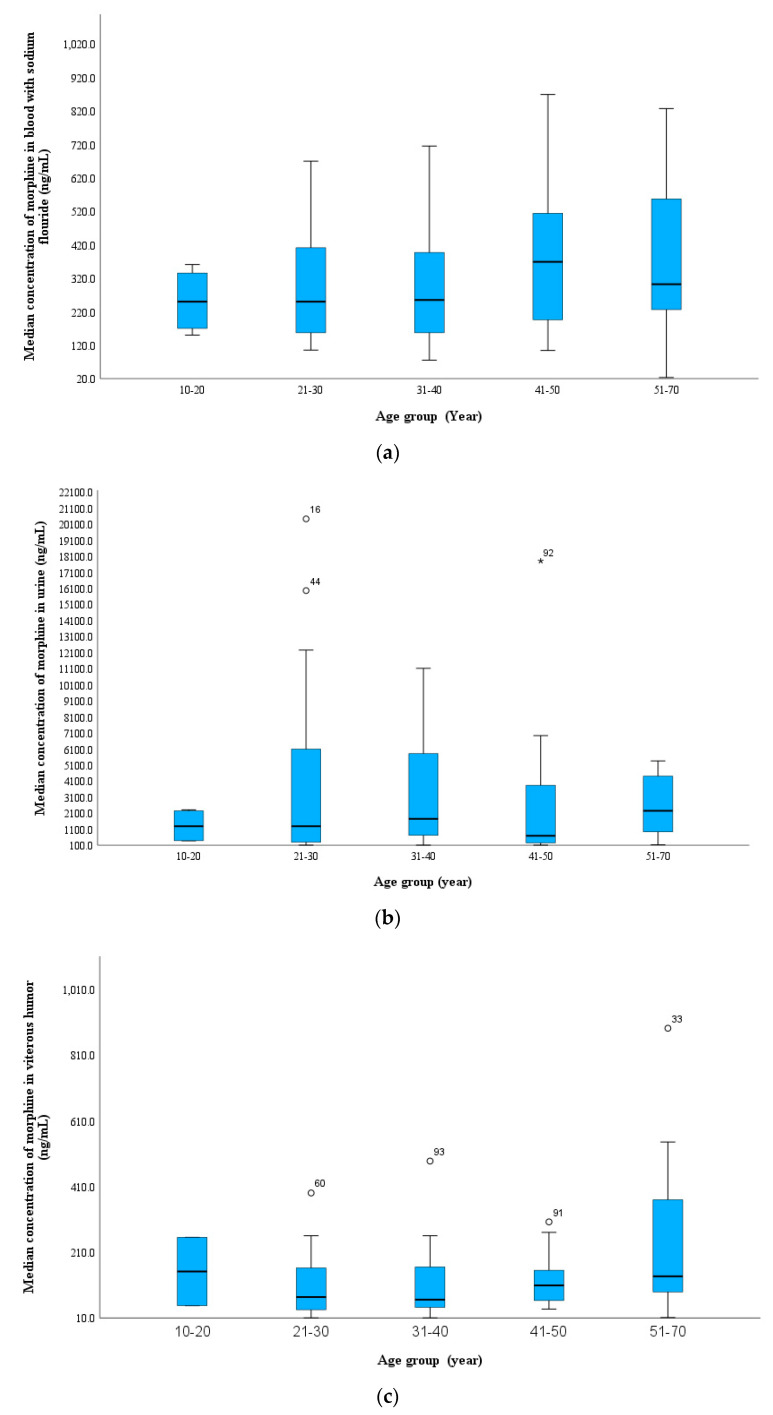

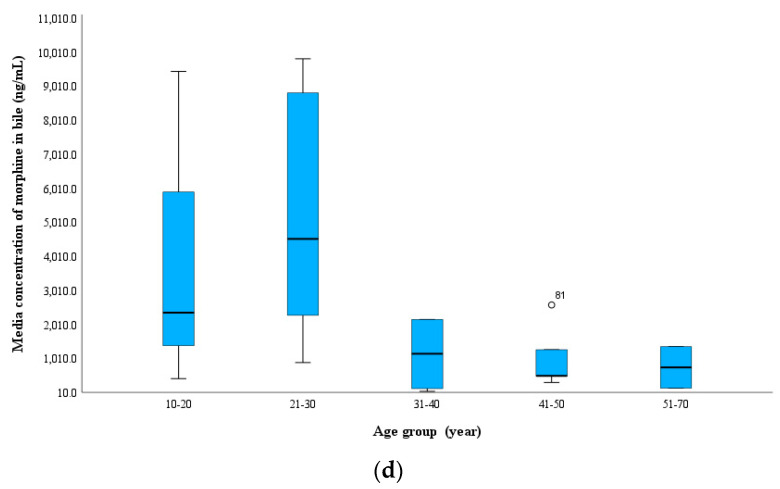
Variation of morphine concentration in the 97 heroin-related fatality cases according to age group (**a**) blood with sodium fluoride (ng/mL), (**b**) urine (ng/mL), (**c**) vitreous humor and (**d**) bile. The whiskers correspond to the smallest and largest value within 1.5 times the interquartile range, and circles (outlier) symbolize values exceeding at least 1.5 times the interquartile range and extremes (asterisks) correspond to values exceeding at least 3.0 times the interquartile range (this description is applied to the remaining box-plot figures).

The mean age of the patients was 38 ± 12 years (range, 16–70 years; males, 98%). The 21–30 age group had the highest number of deaths, with almost 33% of the total cases (n = 32 cases), followed by the 31–40 age group (n = 26 cases, 27%), while the 10–20 age group had the lowest number of heroin-related death cases (n = 4 cases, 4%; Figure 3 and Table S3). Notably, a gradual increase in the median blood morphine concentration occurred from the youngest to the oldest age groups, with a median of 190 ng/mL in the 10–20 group and 302 ng/mL in the 51–70 group. This was also observed for morphine in the vitreous humor; in relation to age, the 6-MAM median concentration showed the same trend as morphine. 6-MAM was not detected in 75% of the cases in the 10–20 year age group, whereas it was present in 40% in the 61–70 year age group and at concentrations (BNaF median, 21 ng/mL; vitreous humor median, 52 ng/mL). This can be understood by the increase in tolerance that occurred as the duration of heroin addiction increased. Therefore, 6-MAM was more likely to be detected in the older age groups than the younger age groups. In contrast, 6-MAM was higher in the biliary specimens of the youngest age groups, as indicated in Figure 3.

In the current investigation, 6-AC was more likely to be detected in the middle-aged groups and was detected in most urine samples in all age groups in this study. This finding indicates that urine is the best choice matrix for 6-AC analysis in both antemortem and postmortem samples [17,30,31]. In relation to age, the codeine concentration in the blood was slightly higher in the older age groups.

#### 3.2.3. PMI and Analyte Concentrations

**Figure 4:**
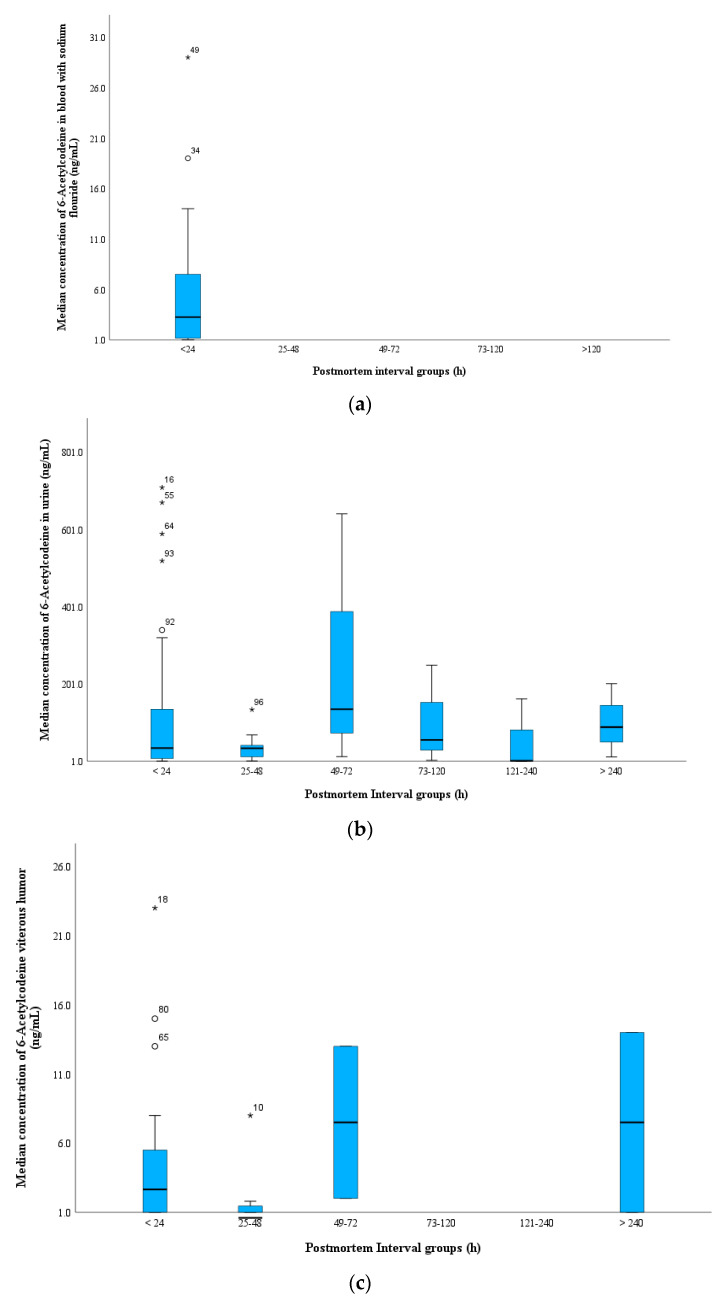

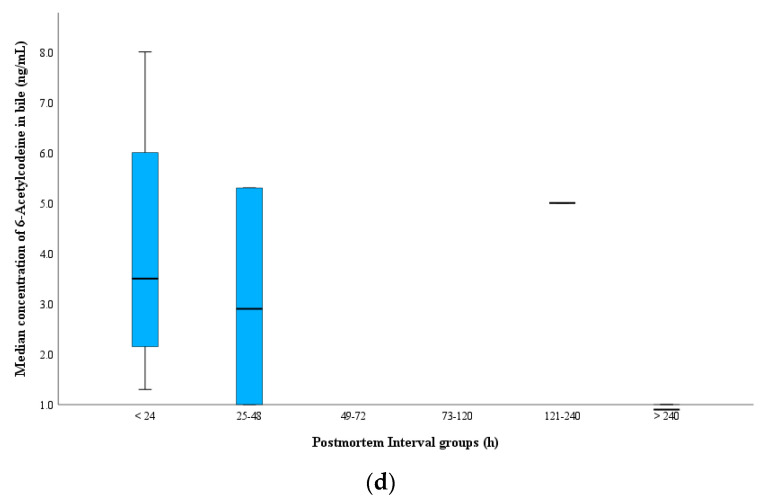
Distribution of 6-AC in the 97 heroin-related fatality cases according to postmortem interval time group (**a**) blood with sodium fluoride (ng/mL), (**b**) urine (ng/mL), (**c**) vitreous humor and (**d**) bile. The whiskers correspond to the smallest and largest value within 1.5 times the interquartile range, and circles (outlier) symbolize values exceeding at least 1.5 times the interquartile range and extremes (asterisks) correspond to values exceeding at least 3.0 times the interquartile range (this description is applied to the remaining box-plot figures).

The majority of cases had a PMI within 24 h (51% of total cases), which led to the identification of both heroin biomarkers when the blood was fresh and putrefaction was not observed. The median concentrations of 6-MAM and 6-AC in cases with a PMI within 24 h were 16 and 2 ng/mL, 22 and 3 ng/mL, 324 and 35 ng/mL, and 12 and 4 ng/mL in the BNaF, vitreous humor, urine, and bile samples, respectively (Table S4). 6-AC was present in the blood at extremely low concentrations in cases where the PMI was less than 48 h, and no 6-AC was detected in any BNaF cases when the PMI was greater than 48 h, although most cases were stored properly following death. In contrast, 6-AC was still detected in the urine samples from all PMI groups (Figure 4). As expected, the median morphine concentration in BNaF increased with longer PMIs. The lowest median morphine concentration (263 ng/mL) was observed at a PMI of 24 h, and the highest concentration (380 ng/mL) was observed at a PMI ranging from 121–240 h (Figure S1).

In the current study, both biomarkers were detected in the vitreous humor in two cases with a PMI longer than 10 days, whereas 6-MAM was negative in the BNaF samples, which indicates that the role of PMIs on heroin biomarkers can be minimized if samples are stored correctly following autopsy and if the vitreous humor is the sample of choice for heroin-related fatality investigations. Figure S2 shows the distribution of 6-MAM in BNaF and vitreous humor samples among PMI groups in the current study.

#### 3.2.4. Mode of Death

In the current study, heroin alone contributed to death in 68% of the studied cases, whereas intoxication with other co-ingested substances contributed to 32% (Table 2). A higher median morphine concentration (310 ng/mL) was observed in the heroin-only cause of death cases than in the polydrug intoxication group (250 ng/mL). The median 6-MAM concentration was similar between these two groups, whereas the median 6-AC concentration in the heroin-alone cases was higher (2.5-fold) than that in the polydrug intoxication group. No difference was observed between the codeine concentrations in the two groups.

These findings indicate that the morphine concentration in heroin-related fatalities showing the presence of other CNS drugs is often lower than that in heroin-alone cases. The significant role of the PMI in cases of heroin metabolites is well known, and the tolerance and health status of the deceased contribute to this finding. Similar trends were observed among the various specimens, with slightly higher median concentrations in the urine and bile. Moreover, a polydrug intoxication case showed a slightly higher morphine concentration in the vitreous humor than that with heroin alone.

The most frequently detected drugs, regardless of the cause of death, were methamphetamine, cannabis, amphetamine, alprazolam, cocaine, and ethanol. Thirty-eight cases only showed heroin metabolites. A higher median morphine concentration was measured in only heroin cases compared to those in which heroin was the sole cause of death despite the presence of another drug, such as amphetamine or cannabis (370 ng/mL vs. 310 ng/mL). Similarly, the median morphine concentration (312 ng/mL) decreased when one extra drug was used, and it was further decreased (191 and 198 ng/mL) when two and four extra drugs were co-ingested, respectively.

Notably, methamphetamine was co-ingested with heroin in twenty-three cases (BNaF median morphine concentration, 284 ng/mL). The median morphine concentration for most co-ingested drugs was often higher than 200 ng/mL. A reduction in the median morphine concentration (BNaF, 154 ng/mL) occurred when heroin was used in combination with cocaine.

#### 3.2.5. Time Span between Heroin Intake and Death

**Figure 5:**
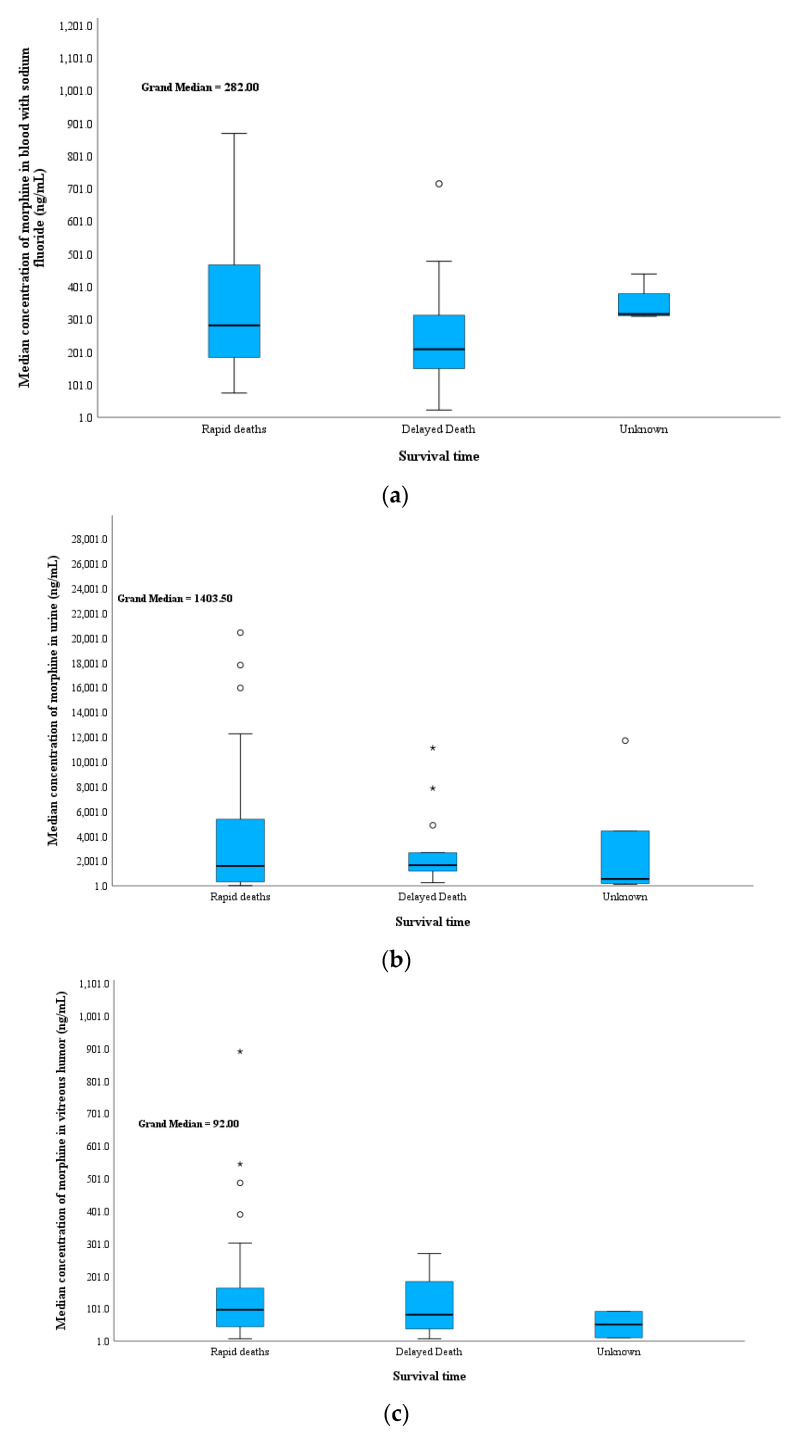

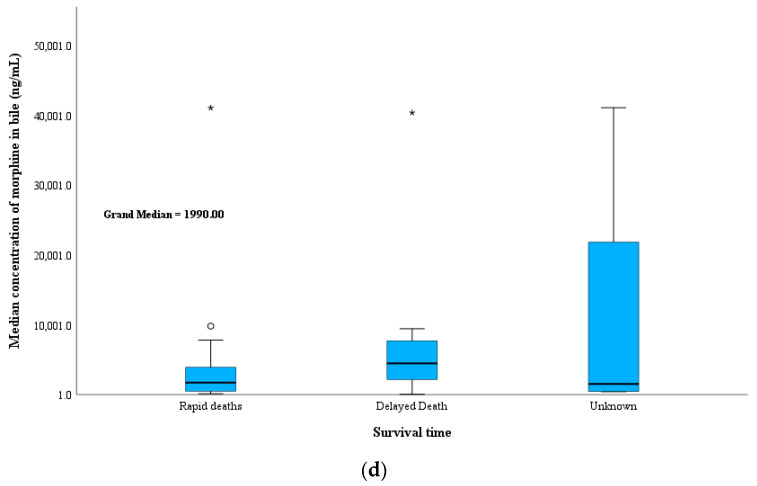
Variation of median morphine concentration in the 97 heroin-related fatalities cases according to survival time before death; (**a**) blood with sodium fluoride (ng/mL), (**b**) urine (ng/mL), (**c**) vitreous humor and (**d**) bile. The whiskers correspond to the smallest and largest value within 1.5 times the interquartile range, and circles (outlier) symbolize values exceeding at least 1.5 times the interquartile range and extremes (asterisks) correspond to values exceeding at least 3.0 times the interquartile range (this description is applied to the remaining box-plot figures).

In this study, 61% and 24% of the cases were rapid and delayed deaths, respectively, and 15% had an undetermined time of death. This was either because of a lack of information caused by the deceased dying without a witness or because the bodies were moved after death to a deserted area where the bodies began to decompose and thus did not show signs of heroin use, such as injection marks (Table 3 and Figure 5). In these cases, both heroin biomarkers tested negative in the BNaF samples. Although a higher PMI was detected in these cases, heroin biomarkers were detected in alternative body fluids, and all urine samples tested positive for 6-MAM.

This suggests that either the undetermined cases died immediately after heroin was administered and the environmental surroundings allowed for the hydrolysis of 6-MAM to morphine or that a delayed death occurred, which allowed morphine glucuronide to deconjugate to free morphine due to the long PMI before sampling. In contrast, lower median morphine concentrations were observed in the delayed death group (median BNaF = 210 ng/mL) than the rapid death group.

In all groups, the median morphine concentration was much lower in the vitreous humor than the blood. This indicates that some of the heroin biomarkers were converted to their metabolites in the blood but were stable in the vitreous humor after death. Nevertheless, the vitreous humor results should be interpreted with caution because a certain amount of morphine is associated with accumulation from the use of old doses by chronic heroin users. Interestingly, 6-MAM levels were higher for the rapid death cases (36 ng/mL) than the delayed death cases (14 ng/mL) and undetermined death cases (7 ng/mL). This can be explained by many factors, including the longer PMI in delayed deaths, putrefaction effects, and body storage effects, especially when the time of death is unknown. These conditions led to a decrease in the 6-MAM concentration in the vitreous humor. This highlights the importance of testing the vitreous humor in cases of longer PMIs and negative 6-MAM in the blood.

Morphine levels in bile were primarily detected in chronic heroin users, which limits the use of these values in distinguishing between rapid and delayed deaths. 6-MAM in bile was detected in 10 of 17 rapid deaths, 3 of 6 delayed deaths, and not detected of 4 undermined cases. Urine is most likely to be positive for heroin biomarkers and their metabolites in these types of deaths. In this study, the free morphine concentration was high for rapid deaths (2100 ng/mL) compared to that for delayed deaths (1300 ng/mL) and undetermined cases (550 ng/mL). An almost 4-fold higher median concentration of 6-AC in urine was observed for rapid death (40 ng/mL) compared with delayed death (10 ng/mL). Moreover, the median codeine concentration was higher in the unknown group (30 ng/mL) than in the rapid (20 ng/mL) and delayed groups (10 ng/mL). Codeine forms quickly after heroin ingestion as a product of 6-AC degradation. Most codeine is metabolized to codeine-6-glucuronide, while some is metabolized to morphine.

#### 3.2.6. Manner of Death

In the current study, 76% of the total cases were ascribed to accidental death, and they had a median age, median BNaF morphine concentration, and PMI of 38 years, 280 ng/mL, and 24 h, respectively. Nevertheless, most heroin abusers in Saudi Arabia used drugs in private or remote areas, such as deserted areas and open land outside the city. In the case of overdoses, deceased bodies left behind without witnesses complicated the identification of the mode of death, especially if the bodies were heavily putrefied.

Information about intentional overdoses was available for seven heroin-related fatalities. Four of these individuals died at home, and two died outdoors while accompanied by a friend. These cases had a median BNaF morphine concentration of 480 ng/mL, a PMI of 24 h, and an age of 48. The highest levels of vitreous humor and morphine were detected in suicide cases (150 ng/m).

In five cases, the mode of death was identified as homicide, and they presented a median BNaF morphine concentration of 180 ng/mL, a PMI of 24 h, and an age of 22. Notably, most cases were discovered outdoors.

The data in Table 4 clearly show that heroin biomarkers were higher in suicides, which had median concentrations of 210 ng/mL for 6-MAM. The role of multiple specimens was crucial for the undetected modes of death because few blood specimens were available for testing. The detection of heroin biomarkers facilitates the source of opioid identification and survival time. In some cases, although syringes and heroin bags were found at the scene, the blood samples were negative for heroin biomarkers. No difference in codeine concentrations was observed according to the mode of death. The manner of deaths was not determined in the last 11 cases.

#### 3.2.7. Route of Administration

Table 5 demonstrates that heroin injection was the main route of administration in the current investigation, followed by sniffing. The route of administration was unknown in 16 of cases, with most involving decomposed bodies. Syringes were found at the scene in 36% of the studied cases. Needle marks were identified in 65% of the total cases, whereas heroin powder was found in 18% of the total cases. Heroin was sniffed in only 9% of cases, which indicates that injection is the main route of heroin administration in Saudi Arabia.

Table 6 indicates that the median morphine BNaF concentration when the drug was administered via injection was 300 ng/mL, followed by sniffing at 150 ng/mL and an unknown route of administration at 220 ng/mL (Table 5). The highest BNaF morphine concentrations (340 ng/mL and 310 ng/mL) were observed when heroin powder and syringes were found near the deceased, respectively. In cases where both syringes and heroin powder were discovered, the median BNaF morphine concentration was 400 ng/mL. No differences were observed in the median vitreous humor concentration among the three routes of administration, with a median of 90–120 ng/mL in all groups. The heroin powder group showed a slightly higher morphine vitreous humor concentration, followed by cases with syringes and heroin bags at 160 ng/mL and 140 ng/mL, respectively (Table S5). In contrast, the median urine morphine concentration was higher in cases of injected heroin (2400 ng/mL) than in the sniffing heroin group (1400 ng/mL). The median bile morphine concentration was almost double in the sniffing group (4200 ng/mL) than in the injection group (1600 ng/mL).

#### 3.2.8. Location of Deaths and Putrefaction

Most heroin deaths occurred in outdoor environments (63%), with a median PMI of 48 h, whereas 37% of the patients died in a private home (Table 6), with a median PMI of 24 h. A higher median BNaF morphine level was observed in those who died indoors (320 ng/mL) than outdoors (240 ng/mL), while the level in vitreous humor was similar (100 ng/mL vs. 80 ng/mL, respectively). In contrast, the median urine and bile morphine concentrations were two- and three-fold higher among those who died outdoors than indoors, respectively.

It has been mentioned that the weather in Saudi Arabia is extremely hot most of the year, and the weather in Jeddah is known to be highly humid. Accordingly, it is well-known that putrefaction and postmortem changes would begin a few hours following death if bodies were not stored appropriately [32,33]. In cases in which heroin was injected and the person dies alone in a closed indoor area, putrefaction will occur faster owing to the combination of hot weather and high humidity in Jeddah city. Considering that most of the deceased die indoors and are discovered in a closed toilet, garage, or hidden area at home, such as the roof, this could facilitate postmortem changes, especially when deaths occur at night with no witnesses. In contrast, a deceased body on the street (outdoor) would be easily discovered unless it was moved to an empty or deserted area.

In 63 out of 97 cases (65%), police and forensic pathologist reports indicated that no witnesses observed the final moments before death (Table S5). Nine patients (11%) were transferred to the hospital; however, most patients died either at the scene before transfer to the hospital or during transfer to the hospital. Identifying the mode of death was difficult due to a lack of information and heavy decomposition of the bodies, especially for those who died outdoors, in the desert, under construction bridges, or in old buildings. In 8 of the 97 cases, the manner of death was unknown due to putrefaction, and these cases presented a median BNaF morphine concentration of 380 ng/mL, a PMI of 230 h, and an age of 40. High morphine concentrations can be explained by the administration of a high dose or due to postmortem changes after death that cause the conversion of 6-MAM and morphine conjugates to free morphine.

In this study, higher BNaF morphine concentrations were measured in patients whose deaths were pronounced at hospitals (n = 9 cases, 450 ng/mL), followed by those found in the street (n = 9 cases, 335 ng/mL), under bridges (n = 11 cases, 262 ng/mL), in cars (n = 17, 240 ng/mL), in rental flats or hotel rooms (n = 7, 237 ng/mL), and in deserted areas (n = 8 cases, 170 ng/mL). PMI, environmental conditions (location of death and weather), and patient tolerance have major effects on the detection of heroin biomarkers and metabolites. The patients who died at the hospital had a lower PMI, and their deaths were caused by an overdose of heroin alone. In contrast, among individuals who died under bridges, most cases showed putrefaction, and 6 out of 10 cases were polydrug intoxication cases. Notably, a high number of deaths occurred in cars, with 13 out of 17 cases of polydrug intoxication having a relatively low PMI (median 24 h) and 6 cases exhibiting signs of putrefaction, with two heavily putrefied. Vitreous humor and urine were available in 13 and 12 of these cases, respectively, and 6-MAM and 6-AC were detected in 6 and 9 cases, respectively. Only one case was positive for both heroin biomarkers in bile. A higher median of BNaF morphine was observed compared to vitreous humor (60 ng/mL), which suggested a shorter survival time. This was supported by the lower urine morphine concentration (860 ng/mL) and highest bile morphine concentration (9800 ng/mL), suggesting a chronic heroin user.

Twenty-one cases showed signs of putrefaction (Table 6), 60% showed partial purification, and 8 out of 21 putrefied cases showed heavy decomposition. There was no difference in age between the two groups; however, the PMIs were higher in the heavily putrefied group (heavy putrefaction: 228 h; partial putrefaction: 96 h; Table 6). 6-MAM was detected in BNaF in two cases in each group. The advantages of testing alternative specimens in determining the source of opioids have been clearly observed in these putrefaction cases because most of the alternative samples, namely urine, vitreous humor, and bile, were positive for 6-MAM. A few cases that showed partial putrefaction tested positive for 6-AC.

#### 3.2.9. Seasonal Distribution

The seasonal distribution of heroin-related fatalities is presented in Table S6 and Figure S3. The highest proportion occurred in spring (29%), followed by summer (28%), winter (27%), and autumn (16%). The highest mortality rate occurred in July (12%), followed by April (11%). The lowest proportion of heroin fatalities (4 %) occurred in October.

#### 3.2.10. Multiple Specimens

The median morphine concentrations in the BNaF, urine, vitreous humor, and bile samples were 280, 1400, 90, and 2200 ng/mL, respectively. Among the available BNaF, urine, vitreous humor, and bile specimens, 6-MAM was detected in 60%, 100%, 99%, and 59%, while 6-AC was detected in 24%, 68%, 50%, and 30%, respectively. The presence of sodium fluoride in the blood test tube as a preservative prevented 6-MAM from converting to morphine in most cases. Additionally, the availability of urine, bile, and vitreous humor samples provides valuable information on the opioids that have been administered. The median free morphine concentration ratios among the vitreous humor/BNaF, urine/BNaF, and bile/BNaF were 0.37-fold (n = 63), 5.6-fold (n = 64), and 7.3-fold (n = 23), respectively. In addition, free morphine/free codeine were always higher than 1, with median ratios of 13-fold (n = 84), 13-fold (n = 70), 6-fold (n = 68), and 85-fold (n = 25) for BNaF, urine, vitreous humor, and bile, respectively.

The Spearman correlation coefficient (R) was used to establish the relationship between heroin biomarkers, morphine, and codeine concentrations in various body fluids (Table S7). 6-MAM in BNaF exhibited a strong positive correlation with the analytes of interest in both the urine and vitreous humor, except for 6-MAM in BNaF vs. 6-AC in the vitreous humor. In contrast, the correlation between 6-MAM in BNaF and analytes of interest in bile was weakly positive for 6-MAM, 6-AC, and codeine but negative for morphine (non-significant *p*-values higher than 0.5). The correlation between the 6-AC concentration obtained from multiple biological fluids was always poor and not statistically significant, and it was not calculated for bile due to the lack of positive samples. A negative correlation was observed between 6-AC and morphine in bile and analytes of interest in the BNaF, urine, and vitreous humor samples, as shown in Table S7. The poor correlation between 6-AC in the different specimens could be explained by the low concentrations, small sample size, and instability of 6-AC in biological specimens. The 6-AC concentrations in BNaF were not significantly correlated with the analytes of interest in the urine and bile, while a significant correlation was obtained with the analytes of interest in the vitreous humor.

Good correlations were obtained between the analytes of interest detected in BNaF vs. vitreous humor, BNAF vs. urine, and vitreous humor vs. urine. The level of 6-AC seemed to be higher in the vitreous humor than the blood, which can directly reflect the anatomical location of the vitreous humor; thus, 6-AC in the vitreous humor is more stable and has a longer half-life before conversion to codeine than that in blood samples.

The bile samples showed the lowest positive results for 6-AC (eight cases) compared to 6-MAM. The 6-MAM was detected in 16 of the 27 bile samples tested in this study. Although the correlation between heroin-related compounds in BNaF and bile was weak, the correlation between these analytes in bile and their corresponding specimens in vitreous humor and urine was strong, except for the 6-AC concentration.

## 4. Discussion

### 4.1. History of Heroin Abuse

In the literature, few studies have detailed the relationship between addiction and morphine concentration. Steentoft et al. reported 245 heroin-related deaths, and all of these cases were from known heroin abusers with the exception of 13 cases [34]. Elfawal pointed out the lack of history of drug abuse in opiate overdose cases in the eastern region of Saudi Arabia [14]. In the current study, a history of heroin abuse was observed in only 24% of all cases. This indicates an improvement in healthcare reporting regarding postmortem drug abuse in Saudi Arabia, considering that the Elfawal study was conducted in 1999 [14].

### 4.2. Age Group and Analyte Concentrations

Heroin users often die young because of polydrug intoxication [35], and most of the deceased are male. Previous reports showed that middle-aged groups and individuals from 21–40 were most likely to be at risk of heroin intoxication, which was demonstrated in the current study based on this group accounting for 60% of the total cases. In one study from Jordan, the mean age of five cases of heroin-related death was 33 [36]. In the eastern region of Saudi Arabia, Elfawal reported that almost 60% of drug-related deaths occurred in individuals aged 20–29 years old [14]. A study from Iran reported that 34% of opioid-related fatalities occurred in individuals aged 20–29 years [37]. In Victoria, Australia, the median ages of opioid-related fatalities were 30 and 29 for males and females, respectively [38]. In Sweden, the median age of heroin-related deaths ranges from 34 to 35 in two different studies [2,39]. An older mean age of 47 has been reported for Minneapolis, MA, USA [40]

Jones and Ahlner studied 766 postmortem heroin-related deaths and 124 traffic antemortem cases related to heroin use and found no correlation between the concentration of free morphine and the age of heroin users (r = 009, *p* > 0.05) [2]. These findings were consistent with those reported by Darke et al. [41]. It is believed that older heroin users likely received higher doses, developed tolerance, and were less likely to change their drug use practices. In the present study, a higher median morphine concentration in the blood was observed in individuals older than 40 compared to those younger than 40, which may be related to the increased tolerance to heroin in long-term abuse cases. A higher 6-MAM level was detected in the blood of the older age group (the highest 6-MAM median blood concentration (250 ng/mL) was found in the 61–70-year age group), while 6-AC was found at low concentrations in all age groups.

The level of codeine was low in all age groups (median, 20–35 ng/mL), which could be explained by the fact that 6-AC is metabolized to codeine and codeine-6-glucuronide (C6G) faster than to other compounds. A recently published study demonstrated that C6G was not detected in blood samples obtained from authentic postmortem cases following heroin intake, while codeine was [42]. In another study, codeine was always higher than C6G in autopsy blood samples from heroin- and codeine-related fatalities [19]. Thus, reporting the free codeine concentrations in the blood is accurate and less laborious. In contrast, C6G was frequently present at a much higher concentration than codeine in many cases attributed to codeine intoxication [43].

### 4.3. PMI and Analytes Concentration

Skopp concluded that the PMI competes with the postmortem redistribution phenomenon (PMR) [44], and they indicated that postmortem changes that occur between death and the discovery of the body include degradation and the formation of drugs or new products. These changes may occur before autopsy and during sample transfer and storage, which is consistent with the many cases of ethanol synthesis after death, for example. Changes that occur during the PMI period and the stability of heroin biomarkers have been well addressed in the literature and can be investigated by assessing the conditions surrounding the cases under investigation, such as the condition of the body after putrefaction has started. A longer PMI can decrease and increase the amounts of heroin biomarkers and their metabolites, respectively, and the presence of these heroin biomarkers cannot be detected with a longer PMI. However, Gerostamoulos and Drummer [45] investigated the influence of PMR on heroin-related fatalities (n = 40, mean PMI = 59 h) and did not observe significant changes in morphine and its metabolite concentrations between antemortem and postmortem samples. This observation was supported by the work of Logan and Smirnow, who also found no significant difference in free morphine concentrations obtained from different anatomical sites (n = 32 cases) [46]. In 19 cases related to codeine intoxication, they found that the PMI did not have a significant effect on the production, formation, or redistribution of codeine-related metabolites, including morphine and its metabolites [43].

Fugelstad et al. studied the effects of postmortem changes on heroin-related fatalities and concluded that the concentration detected at autopsy was the same as that at the time of death [39]. In contrast, Sawyer and Forney studied the effect of different PMIs on the concentration of morphine in rats and found that the morphine metabolites increased by almost 300% [47]. This increase may be due to the fast release of fluids from tissues following death in small animals, which leads to increased morphine concentrations in postmortem body fluids [45,46]. Although morphine concentrations differed depending on the site of collection, such as cardiac blood and femoral blood, this can be due to the incomplete distribution of the drug after death and mostly represents anatomical site-to-site differences, which should be considered when interpreting heroin-related fatalities [4,45,48].

PMR has been debated scientifically, especially for postmortem blood morphine concentrations following heroin-related deaths. Maskell et al. suggested the use of vitreous fluid to verify heroin use because minimal morphine change was detected in their study [49]. An increase in free morphine after death can be expected due to the conversion of 6-MAM to morphine; in some cases, morphine-conjugated degradation can be expected, especially during the PMI period [2,50,51]. Including multiple specimens is crucial for identifying the source of the opiates used. In the case of vitreous humor, this study showed that 6-MAM is higher than BNaF in this fluid and free morphine is lower than BNaF. Similar observations were reported by Scott and Oliver [52] and Rees et al. [51]. Different results have been obtained for urine samples because low free morphine concentrations should be expected due to morphine-conjugated formation before excretion into urine [53,54]. Heroin biomarkers were more stable in vitreous fluid than in blood [55], which is supported by the detection of high 6-MAM in vitreous humor in cases with a longer PMI, whereas 6-MAM was negative or found at a low level in BNaF samples. The probability of obtaining a vitreous humor specimen for testing decreases with an increase in the time between death and body discovery. In addition, longer PMIs decreased the likelihood of obtaining such specimens unless the body was stored properly immediately after death [33]. The combination of BNaF and vitreous fluids or BNaF and urine analysis are crucial tools for identifying the source of morphine in postmortem cases with a PMI greater than 48 h. Bile is a unique matrix for assessing the chronic use of drugs [20], and it was tested in only 27 cases in the current investigation. Interestingly, both heroin biomarkers were detected in all PMI period groups; however, due to the small sample size in the long PMI groups, we could not examine the correlation between BNaF and bile when assessing heroin-related deaths. However, the presence of heroin biomarkers might be a potential indicator of a recent, rapid overdose of heroin, regardless of the PMI period after death. Codeine is formed by 6-AC degradation, and most of the 6-AC is completely converted to codeine following injection [2]. The highest median codeine concentration in the current study was observed with a PMI of 73–120 h after death (BNaF: 50 ng/mL, vitreous humor: 20 ng/mL).

### 4.4. Mode of Death

The use of blood-free morphine/total morphine ratios in the blood to identify the cause and mode of death has been reported [18,42]. However, this approach always involves certain issues. For example, morphine3-glucuronide and morphine-6-gluccronide are known to be unequal in terms of production and potency, and sample preparation using different hydrolysis procedures (acidic or enzymatic) seems to differ depending on the individual laboratory setting. The stability of morphine conjugates is affected by the phenomena of redistribution following death and enterohepatic recirculation. The total morphine content can be affected by the accumulation of morphine from previous injections. Therefore, free morphine is more important than total morphine for determining the blood concentrations of heroin-related fatalities [2,20,56].

Jones and Ahlner found no differences in the median blood morphine concentration between intoxication with polydrug and heroin-only [2]. Meissner et al. found that the median free morphine concentration was higher in polydrug intoxication than in heroin intoxication alone (232 ng/mL vs. 170 ng/mL, respectively) [57]. Al-Asmari reported that the median free morphine in heroin-only intoxication cases was marginally higher than that of multiple drug intoxication cases (152 vs. 108 ng/mL, respectively) [20].

This study is consistent with a previous investigation that found lower morphine concentrations when cocaine was co-administered [58]. The low free morphine concentration in blood in polydrug intoxication cases is consistent with a previous investigation that suggested that the possibility of overdose was potentially increased with the presence of other CNS drugs, even with low free morphine concentrations, due to the cumulative effects of these drugs, including heroin [59]. In contrast, in the presence of both cocaine and heroin, a higher free morphine concentration was obtained because both drugs are metabolized by carboxylesterases [58], which may inhibit the formation of morphine conjugates [19].

In this study, few case were positive for heroin and ethanol, where ethanol was attributed to antemortem ethanol ingestion. Moreover, the few cases may have been related to the low sample size of heroin users and the prohibition on ethanol intake by law in Saudi Arabia due to religious reasons [11,60]. In contrast, 28% and 13% of heroin cases showed co-ingestion of cannabinoids and amphetamine, respectively, which are more popular drugs in this region [25]; however, these drugs rarely contribute to death [61,62,63]. 6-MAM was detected in BNaF in most cases that tested positive for antemortem ethanol, which is consistent with the results of previous investigations [19].

Notably, Figure 2 shows the median concentration of morphine per year. In 2018, there were more cases but much lower concentrations than in 2014. The highest morphine concentration was observed in the current study despite having the fewest cases, and this can be interpreted as follows. In 2014, most cases involved mono-heroin intoxication, and almost 60% of cases were putrefied, which explains the high concentration of BNaF. In contrast, cases in 2018 comprised polydrug intoxication, including methamphetamine, cocaine, and tramadol, and only 20% of cases presented with slight putrefaction. In this project, a shift from mono-intoxication to intoxication from 2008, when this project was started, to a poly-drug intoxication trend at the end of this report in 2018, was observed.

In 2018, alprazolam was detected in 4 out of 15 cases; most of these cases involved polydrug intoxication, including methamphetamine, cocaine, and tramadol. In Saudi Arabia, alprazolam is a controlled drug, although the alprazolam supply has increased in illegal markets, which explains this increase. A similar trend has been observed with methamphetamine-related postmortem deaths in Jeddah, Saudi Arabia, in the same period [64]. Abuse of alprazolam and heroin has been previously reported, which reflects a high number of prescriptions or supplies from the illegal market [65]. In addition, few deaths related to alprazolam have been reported in the literature; however, intoxication cases with alprazolam and other drugs, especially cocaine, have been reported [66].

### 4.5. Time Span between Heroin Intake and Death

Consistent with previous reports, deaths following heroin intake can be divided into three types: rapid and delayed deaths, and undetermined survival times [2,3,51]. Rapid deaths can be identified by the detection of 6-MAM in blood samples, fresh needle marks, and the presence of syringes attached to the deceased body or nearby [3,42,59]. In addition, valuable information on the last moment before death can be obtained from family, friends, or witnesses [13]. Negative blood samples for 6-MAM may indicate a longer time between death and heroin administration (delayed death), which is supported by the detection of 6-MAM in other specimens, such as vitreous humor, urine, and bile [20,67]. Rapid deaths were expected to occur within <3 h after administration based on the detectable 6-MAM in blood, while delayed deaths occurred >3 h after administration based on the absence of 6-MAM in blood but presence in other specimens [3,51,68,69].

Goldberger et al. reported similar findings to the current study and showed that a higher median free morphine concentration occurred for rapid deaths (1420 ng/mL) compared to delayed deaths (250 ng/mL), while the median morphine for undetermined survival time after ingestion (610 ng/mL) was higher than that for delayed deaths [70]. Recently, Jakobsson et al. reported on 35 acute heroin-related deaths and found that the median free morphine concentration was 350 ng/mL for rapid deaths and 130 ng/mL for delayed deaths, respectively. The authors suggested that this discrepancy can be ascribed to a high heroin dose in rapid deaths [42]. This hypothesis is supported by the results from the current study and a previous investigation by Dark et al., who found that the median free morphine in rapid heroin death cases (260 ng/mL) was double that of delayed death cases (120 ng/mL) [58]. In addition, in some undetermined cases, the bodies were discovered many days after death, which increased the time between death and analysis. Such extended time periods were sufficient to convert 6-MAM and morphine conjugates to free morphine, which led to increased morphine concentrations in the blood. In contrast, in cases of delayed death, a considerable amount of morphine was eliminated before death, which resulted in low morphine concentrations [3].

The concentration of morphine in the vitreous humor is often lower than that in the blood. However, this concentration was found to be dependent on the survival time following heroin administration.

The use of the bile free morphine concentration to distinguish between rapid and delayed deaths has received little attention, and in most cases, the total morphine content has been reported. The total morphine concentration is significantly higher with delayed deaths, which indicates that the deceased is heroin-dependent and has received high doses of heroin [70]. The same observation was obtained in the current study, in which the bile free morphine concentration was higher in delayed death cases (2800 ng/mL) than in rapid (2200 ng/mL) and undetermined cases (1400 ng/mL). A similar finding has been reported regarding the role of urinalysis in cases of heroin-related deaths, with negative morphine or low concentrations used as an indicator of recent or naïve heroin use [71].

### 4.6. Manner of Death

In general, heroin-related deaths are most likely to occur due to accidental overdoses, although suicidal or homicidal modes of death have also been reported in the literature [13,69]. Abiragi et al. stated that identifying the mode of death in these cases is complicated if a relevant history of drug addiction and information on the last moment before death obtained from family or friends are not available at autopsy and in related toxicology reports [13]. One common finding was the lack of available information for individuals who were found dead without witnesses to the last moments before death. Gerostamoulos et al. [38] studied 434 heroin-related deaths in Victoria and found that 67% of these cases were found to be deceased, and 59% of the cases died alone. In that report, ambulances were called for 72% of the heroin-related deaths. Thaulow et al. studied 51 heroin-related deaths, and 64% of cases were found dead alone [3].

Depression is an indicator associated with heroin abuse, and it has led to an increase in violence-related deaths among heroin users [41,72]. Darke and Ross reported that the suicidal manner of heroin-related deaths ranges from 3% to−35% and suggested that heroin users are more prone to die by suicide at a rate 14-times higher than that of non-heroin users [72].

Opioids, such as heroin, are sedative drugs that are rarely associated with violence [72,73,74]. In cases where violence was attributed to heroin, it was considered indirect and not related to the effect of heroin ingestion; for example, it can arise from actions performed to obtain money for purchasing drugs [74]. A direct effect of heroin on homicide can be expected in the case of injecting or spiking heroin to sedate a victim because the dose may be higher than the victim can tolerate, or the user may be naive [74,75,76]. In addition, homicide deaths can occur between drug dealers, and violence also occurs between dealers and users [13,35,74]. Suicide-associated heroin deaths are underreported because it is difficult to differentiate between suicidal and accidental deaths based on the lack of necessary information from family and friends who may have witnessed the last moments of the deceased’s life [13,72].

The median morphine concentration reported in this study was considered higher in cases of suicide-related deaths compared to those of accidental intoxication, which is consistent with the study by Darke et al. [41]. In most suicide cases, the method was a heroin overdose; deaths occurred in closed areas (home or prison); the deceased were male; and unemployment was a factor, which is consistent with prior investigations [72]. Moreover, the long history of heroin abuse reported in these cases may have increased the risk of depression, which led to suicidal thoughts. The highest morphine concentration reported in this study was for the suicide cases, which was consistent with the high blood morphine concentration (>1000 ng/mL) reported in a suicide case [41]. In another study that reported on two suicides by heroin injection, the blood morphine concentration was 630–640 ng/mL [77].

Heroin overdose as a means of homicide has been reported, and the percentages of heroin-related homicide have varied between studies. For example, in a study by Denninng et al., almost 20% of cases were reported to be homicides by drug overdose [78]. In other reports, ≥50% of homicide cases were due to the use of heroin as a means of intoxication [72,79,80,81]. In the current report, homicides were more frequent among younger users (median age: 22), while suicides were more frequent in older users (median age: 48), which is consistent with a previous report by Lee et al. [35]. Homicides cases frequently involved multiple drugs, and in the current study, all homicides cases were positive for cannabis, cocaine, tramadol, and alprazolam which is consistent with the findings of Lee et al. [35]. In contrast, suicide-related heroin cases mostly involve heroin alone. The most common cause of death is old age, which frequently involves other diseases due to prolonged heroin use, such as HIV and hepatitis, bronchopneumonia/acute pneumonia, cardiac problems, and a history of mental health problems [82,83]. Owing to the above-mentioned lack of drug abuse history and dying in deserted areas, which leads to putrefaction, an undetermined manner of death was recorded in 11% of the current cases. The manner of death in these cases was unknown due to a lack of information, and recent injection markers could not be found. Moreover, identifying the last injection site was difficult because most heroin addicts inject many times a day and most of the bodies were heavily decomposed. 6-MAM was only detected in blood samples from two of these cases.

### 4.7. Route of Administration

Our findings were consistent with the study by Thiblin et al. (median BNaF for injection = 230 ng/mL, non-injection = 100 ng/mL, and unknown routes of administration = 180 ng/mL) [84]. A higher median morphine concentration is often associated with heroin injection compared to non-injection routes. Consistent with the current investigation, Hadidi et al. concluded that the main route of heroin administration in Jorden was injection [36]. Crandall et al. [85] observed recent needle puncture marks in 77% of opioid overdose deaths, whereas drugs were found nearby in 51% of deaths. Darke and Duflou [58] reported that 44% of their cases died immediately, and needles or tourniquets were still attached to the bodies. In that study, 13% of the patients were transferred to the hospital. Thaulow et al. studied 51 heroin-related deaths, and syringes were discovered nearby the bodies in 16 out of 51 cases, with nine and seven classified as rapid and delayed deaths, respectively [3]. In a study from Thailand, needle puncture marks were observed in only 35% of deceased patients [86].

In the current study, no differences were observed in the median vitreous humor concentration among the three routes of administration. The most useful information obtained by measuring vitreous humor is the source of opiates administered when 6-MAM is detected in this matrix [4]. In contrast, Thaulow et al. [3] stated that the interpretation of the vitreous humor concentration of morphine can be complicated by many factors that make its use in postmortem investigations less suitable. One of the most obvious is the accumulation of morphine from a previous injection, especially for chronic users. In addition, it seems that the transport of morphine from the blood to the vitreous humor is slower, and a lag in distribution between these two matrices was reported [51]. Therefore, the use of morphine/codeine ratios to assess the source of opiates administered is not recommended. In the same report, using vitreous humor results, two cases were classified as codeine use if no 6-MAM was detected in the vitreous humor [3]. This is also complicated because the use of prescribed opiates and heroin together can increase this ratio and lead to misleading results. Therefore, no differences between different routes of administration can be expected due to the aforementioned factors. Darke and Ross reported a comparison between injection and non-injection in fetal heroin overdose cases. They found that a lack of differences between these two routes of administration and deaths due to heroin is not restricted to the injection of heroin and non-injection routes, along with the same heroin-overdose threat observed [87].

In a study by Fugelstad et al. [39], patients tested positive for HIV and hepatitis in 7.3% and 88% of cases, respectively. In a study from Victoria, 61% of the total cases tested positive for hepatitis, and only two cases were positive for HIV [38]. In another study, 50% of heroin-related deaths tested positive for hepatitis, and HIV was detected in 2% of the total cases [88].

It has been widely reported that heroin addiction is one of the main causes of HIV, and therefore, we believe that it is important to discuss the number of individuals who died from HIV. As the discussion here pertains to an epidemiological investigation in certain parts of the world for the first time, this piece of information is important. However, one of the limitations of this study is that the infectious disease history was not detailed in all cases. HIV and hepatitis were reported in fewer than 1% and 3% of total cases, respectively, which may indicate that sharing needles is not a common practice or that HIV deaths related to heroin addiction are under-reported in Saudi Arabia, considering that deaths due to infectious diseases are not usually subjected to postmortem analysis.

### 4.8. Location of Deaths and Putrefaction

The location where the bodies were found following a drug overdose is an important factor when interpreting the cause and manner of death. The impacts of ecological conditions, such as temperature and humidity, and location, such as indoor and outdoor places, on the analytes of interest in the postmortem samples were investigated. Few studies have discussed the location of heroin-related deaths. Fugelstad et al. [39] found that one-third of their cases occurred in private homes, almost 60% died alone with no witnesses, and only 9% were witnessed by others. In addition, Gerostamoulos et al. reported 434 heroin-related deaths in Victoria and showed that 60% occurred in the home [38]. In a study from Thailand, 40% and 24% of heroin-related deaths occurred in homes and hotels, respectively [86]. The risk of overdose increases when heroin is administered in public locations because the heroin users may inject the drug rapidly to avoid being noticed [88].

In previous studies, although putrefaction was reported, limited information regarding heroin-related metabolites was available. In the study by Crandall et al., putrefaction was observed in 12% of heroin-related deaths [85]. Crump et al. found that putrefaction degradation products did not interfere with morphine and codeine analyses [89]. Soravisut et al. concluded that morphine was detected in 50% of decomposed bodies in alternative specimens, such as liver tissues [86]. Reisinger et al. [90] compared oral cavity specimens with blood, urine, and bile specimens in cases of heroin-related fatalities. Some of these cases were partially or heavily decomposed (PMI 2–10 days). In their study, 6-MAM was found in the blood or urine of putrefied cases in 6 of 11 cases, which is consistent with the current investigation.

### 4.9. Seasonal Distribution

The impact of seasonal variation on heroin biomarkers and their metabolite analysis is believed to be a crucial factor underlying heroin-related deaths, especially in countries such as Saudi Arabia, where hot weather is more dominant. Kringsholm et al. [91] reported 205 heroin deaths and found that seasonal variations were not significant in most of the tested specimens. However, the weather in Denmark is cold [91] compared to that of Saudi Arabia. The detection of 6-MAM and 6-AC in bile seems to be seasonally dependent, despite the low sample size for bile, and the most frequent detections of these metabolites occurred in January and February. This can be attributed to stability issues for heroin biomarkers during the winter in Saudi Arabia. Soravisut et al. studied 142 opiate-related fatalities and found that the highest number of overdose deaths occurred in May [86].

### 4.10. Multiple Specimens

In forensic postmortem procedures, the ratio of morphine to codeine is of paramount importance in differentiating the source of opioid ingestion. A morphine-to-codeine ratio of less than one is considered an indicator of the intake of medicine containing codeine, while a morphine-to-codeine ratio of more than one is related to the use of heroin [3,51,92]. The interpretation of morphine/codeine ratios in the blood becomes difficult if morphine and heroin are administered. It is recommended to use these ratios in cases where no 6-MAM is detected; moreover, heroin intake can be confirmed if this ratio is higher than one and supported by information from the scene and autopsy findings [3,51]. Berg-Pedersen et al. studied the codeine/morphine ratio under different storage conditions and found that codeine and its glucuronide are more stable than morphine and its glucuronide in antemortem and postmortem cases [93]. Skopp et al. found that morphine conjugates were stable in the blood if they were subjected to proper storage at −20 °C; however, increases in morphine were observed with heroin and codeine intoxication [94]. Morphine-3-glucuronide and morphine-6-glucuronide are cleavage products of morphine, and this cleavage occurs during sample storage, which increases the morphine concentration and affects the ratio between morphine and codeine [95,96]. In this study, the free morphine/codeine ratios were higher than 11, which agrees with previous investigations on heroin-related administration [20,97].

Many factors that affect the connections between heroin biomarkers and their metabolites must be taken into consideration, such as the history of abuse, concomitant use of other drugs, tolerance, route of administration, and PMI [3,33,44]. Mercurio et al. [98] studied 52 morphine-related deaths in which blood and bile were assessed in parallel and found that bile/blood morphine concentrations were poorly correlated (*r*^2^ = 0.01, *p* = 0.4), which agrees with the current investigation. In that study, the median ratio of free morphine between the bile and blood was twenty-nine, which was lower than that in the current study. Duflou et al. reported the total morphine in blood and bile, and a strong correlation for the total morphine between blood and bile was obtained when using their data, with Rs = 0.712 and a *p*-value of 3 × 10^−5^, and the median ratio of free morphine between bile and blood was 37 [96]. The morphine concentration in bile and urine was utilized to investigate whether the deceased had a history of heroin use, and in a previous study, heroin-related deaths were compared between two cities in the USA. In that study, blood morphine concentrations were similar, and both bile and urine provided information that users in San Francisco (n = 27 cases) were heroin-dependent while those in Connecticut (n = 15 cases) were naive or had stopped using heroin for some time before the last injection [54]. Negative or low concentrations of morphine in urine can be expected with rapid deaths related to heroin; for example, in two suicide-related heroin overdoses, the total morphine ranged from 100 to 500 ng/mL [77]. A low morphine concentration <1000 ng/mL is considered an indicator of a short survival time following heroin injection [71]. In addition, Jakobsson et al. compared heroin-related metabolites in urine with the findings of previous reports using free morphine and concluded that a higher concentration of urine free morphine is an indicator of a higher heroin dose [42].

The correlation of morphine in the blood with the vitreous humor has been widely studied. Scott and Oliver [52] found that this correlation was dependent on the rate of death and reported a good correlation (r^2^ = 0.697) in all cases, whereas a strong correlation was obtained in 17 cases when the deaths were described as sudden (r^2^ = 0.885). The same observation was reported by Rees et al. [51], who found a significant correlation for free morphine between the blood and vitreous humor, with Rs = 0.81 for rapid death and Rs = 0.71 for delayed death. In the current investigation, rapid death cases showed a better correlation (Rs = 0.650, *p* = 8.0 × 10^−7^) than delayed death cases (Rs = 0.450, *p* = 0.1).

## 5. Conclusions

To the best of our knowledge, this is the first epidemiological study evaluating heroin-related deaths in Jeddah, Saudi Arabia. Although the weather in Saudi Arabia is extremely hot most of the year, 6-MAM hydrolysis to morphine is favored. The detection of heroin biomarkers (6-MAM and 6-AC) is facilitated by short PMI periods in the majority of cases; moreover, the addition of sodium fluoride as a preservative prevents 6-MAM from being degraded to morphine in most cases. In addition, the availability of urine, vitreous humor, and bile samples provides valuable information on which opioids have been administered based on the detection of heroin biomarkers. The latter is important if no blood samples are available, if blood samples are negative for heroin biomarkers, and in cases of partially and heavily decomposed bodies. The incidence of heroin deaths in Jeddah remained stable during the study period. Most individual deaths likely occurred within groups, but the body was left behind or moved owing to fear among users to call an ambulance. Although only 24% of the deceased had medical records, the findings indicated an improvement in health reporting regarding drug abuse in Saudi Arabia. It is highly recommended that heroin users be encouraged to contact ambulances and trained in resuscitation. This study concluded that most patients were unemployed (up to 53%), had started taking heroin in their teens, and had been admitted several times to the addiction center. Therefore, the loss of tolerance following detoxification might have been the primary cause of many heroin-related deaths.

## Figures and Tables

**Table 1:** Liquid chromatography electrospray ionization tandem mass spectrometry (LC-MS/MS) data for heroin biomarkers, morphine, and codeine.

Analytes ^&^	Internal Standards	RT * (min)	Quantifier Ion	Qualifier Ion	RT (min)	Quantifier Ion	Qualifier Ion
		LC-MS 8050			LCQ Fleet		
Analytes							
6-MAM	6-MAM-d3	6.9	m/z = 328−165	m/z = 328−221	15.1	m/z1 = 328−211	m/z = 328−268
6-MAM-d_3_ ^#^	-	7.0	m/z = 331–165	m/z = 331–221	15.1	m/z = 331–165	m/z = 331–221
6-AC	Codeine-d3	9.3	m/z = 342–225	m/z = 342–165	19.0	m/z = 342–225	m/z = 342–282
Morphine	Morphine-d3	4.7	m/z = 286–165	m/z = 286–153	8.4	m/z = 286–201	m/z = 286–229
Morphine-d_3_ ^#^	-	4.6	m/z = 289–165	m/z = 289–153	8.2	m/z = 289–201	m/z = 289–229
Codeine	Codeine-d3	6.8	m/z = 300–165	m/z = 300–44	13.9	m/z = 300–215	m/z = 300–243
Codeine-d_3_^#^	-	6.7	m/z = 303–165	m/z = 300–199	13.9	m/z = 303–215	m/z = 300–243

^&^ Analytes: 6-monoacetylmorphine (6-MAM), 6-acetylcodeine (6-AC), RT *: Retention time. ^#^ Internal standard.

**Table 2:** Heroin biomarkers, morphine, and codeine concentration in relation to cause of death in the current study.

		Cause of Deaths							
		Heroin Only				Poly Drug Intoxication			
	Total case number	66				31			
		*N*	Med	Min	Max	*N*	Med	Min	Max
Specimens	Analytes ^&^		ng/mL				ng/mL		
Blood with Sodium Fluoride	6-MAM	36	10	1	420	14	10	Tr	100
	6-AC	11	5	Tr	30	9	2	Tr	10
	Morphine	57	310	100	4400	27	250	23	670
	Codeine	57	20	4	140	27	20	3	110
Urine	6-MAM	46	330	1	18,900	28	320	1	4600
	6-AC	30	40	1	670	20	40	1	710
	Morphine	46	1340	90	50,400	2	1850	10	20,400
	Codeine	43	210	5	12,110	27	170	3	1210
Vitreous Humor	6-MAM	45	30	1	240	25	20	1	210
	6-AC	18	2	Tr	10	17	2	Tr	20
	Morphine	45	90	10	890	26	90	10	490
	Codeine	44	10	3	140	25	10	1	70
Bile	6-MAM	9	10	1	160	8	10	2	40
	6-AC	3	5	1	10	5	3	1	5
	Morphine	18	2400	40	41,100	10	1700	300	4
	Codeine	15	20	5	230	10	40	10	120

^&^ Analytes: 6-monoacetylmorphine (6-MAM), 6-acetylcodeine (6-AC). *N*: number of cases; Med: Median; Min: Minimum; Max: Maximum; Tr: Trace concentration.

**Table 3:** Heroin biomarkers, morphine, and codeine concentration in relation to survival time following heroin administered in the current study.

		Survival Time											
		Rapid Deaths				Delayed Death				Unknown			
	Total case number	69				19				9			
Specimens	Analytes ^&^	*N ^#^*	Median	Minimum	Maximum	*N*	Median	Minimum	Maximum	*N*	Median	Minimum	Maximum
Blood with Sodium Fluoride	6-MAM	48	10	Tr	420	0				0			
	6-AC	19	3	Tr *	30	0				0			
	MOR	66	310	80.0	4400	14	210	23.0	715	4	317	310.0	439
	COD	66	20	3.0	140	14	10	3.0	40	4	30	4.0	60
Urine	6-MAM	54	380	1.0	18,876	14	380	10.0	820	6	50	1.0	130
	6-AC	37	40	1.0	710	12	10	1.0	320	0			
	MOR	54	2100	14.0	50,401	14	1300	244.0	11,100	6	550	120.0	11,700
	COD	51	210	3.0	12,110	13	90	3.0	630	6	20	5.0	660
Vitreous Humor	6-MAM	50	40	1.3	240	18	10	3.0	40	2	n.a.	1.0	13
	6-AC	25	3	Tr	20	9	1	Tr	14	1	n.a.	1.0	1
	MOR	51	90	10.0	900	18	60	10.0	270	2	n.a	11.0	90
	COD	50	10	1.0	140	17	10	3.0	70	2	n.a.	2.0	10
Bile	6-MAM	10	10	2.0	160	4	10	10.0	40	3	10	2.0	40
	6-AC	4	2	Tr	5	3	5	4.0	10	1	n.a	1.0	1
	MOR	17	2200	30.0	41,100	6	2800	40.0	9,400	4	1520	410.0	41,100
	COD	16	30	5.0	190	5	40	Tr	230	4	40	10.0	170

^&^ Analytes: 6-monoacetylmorphine (6-MAM), 6-acetylcodeine (6-AC); MOR: morphine; COD: codeine. *^#^ N*: number of cases; * Tr: Trace concentration.

**Table 4:** Heroin biomarkers, morphine, and codeine concentration in relation to manner of death in the current study.

		Manner of Deaths															
		Accidental				Suicidal				Homicidal				Undetermined			
Total Case Number		74				7				5				11			
Specimens	Analytes ^&^	*N* ^#^	Median	Minimum	Maximum	*N*	Median	Minimum	Maximum	*N*	Median	Minimum	Maximum	*N*	Median	Minimum	Maximum
			ng/mL				ng/mL				ng/mL				ng/mL		
Blood with Sodium Fluoride	6-MAM	39	10	Tr *	150	6	210	5.0	420	2	6	2.0	10	3	3	1.0	20
	6-AC	15	2	Tr	10	5	10	4.0	30	0				0			
	MOR	67	280	20.0	830	7	480	220.0	4400	3	180	150.0	870	7	210	120.0	720
	COD	67	20	3.0	110	7	30	5.0	140	3	10	4.0	50	7	10	4.0	40
Urine	6-MAM	57	310	1.0	4600	6	810	30.0	18,900	5	480	54.0	2520	6	540	40.0	1580
	6-AC	36	36	1.0	710	5	40	13.0	670	4	40	1.0	250	5	10	1.0	160
	MOR	57	1330	10.0	20,400	6	3630	100.0	50,400	5	2100	1240.0	5350	6	990	190.0	4110
	COD	54	140	3.0	1300	6	240	5.0	12,110	4	540	170.0	1210	6	150	10.0	2100
Vitreous Humor	6-MAM	57	20	1.0	200	5	210	60.0	240	3	20	3.0	30	5	20	1.0	50
	6-AC	28	2	Tr	20	5	3	1.0	10	1	10	10.0	10	1	1	1.0	1
	MOR	57	80	10.0	890	5	150	40.0	550	3	50	40.0	150	6	120	40.0	300
	COD	55	10	1.0	140	5	20	10.0	30	3	10	10.0	10	6	10	3.0	20
Bile	6-MAM	9	10	2.0	40	1	10	10.0	10	2	30	10.0	40	5	2	1.0	160
	6-AC	5	3	1.0	5	0				2	4	Tr	10	1	5	5.0	5
	MOR	16	2100	40.0	41,100	2	310	130.0	490	3	1350	1260.0	9440	7	2807	120.0	41,100
	COD	13	40	10.0	170	2	20	10.0	20	3	70	5.0	230	7	10	10	190

^&^ Analytes: 6-monoacetylmorphine (6-MAM), 6-acetylcodeine (6-AC); MOR: morphine; COD: codeine. *^#^ N*: number of cases; * Tr: Trace concentration.

**Table 5:** Comparison of different routes of administration on heroin biomarkers, morphine, and codeine concentrations in the 97 heroin-related fatalities cases in Jeddah, Saudi Arabia between 2008–2018.

		Route of Administration											
		Injection				Sniffing				Unknown			
Total Case Number		72				9				16			
Specimens	Analytes ^&^	*N ^#^*	Median	Minimum	Maximum	*N*	Median	Minimum	Maximum	*N*	Median	Minimum	Maximum
Blood with Sodium Fluoride	6-MAM	41	10	1.0	420	5	4	Tr	10	4	5	1.3	20
	6-Ac	17	4	Tr *	30	3	3	Tr	10	0			
	Morphine	63	310	20.0	4400	8	150	75.0	360	13	220	120.0	720
	Codeine	63	20	3.0	140	8	10	3.0	40	13	20	10.0	70
Urine	6-MAM	53	470	1.0	18,900	9	280	20.0	480	12	75	1.0	2520
	6-Ac	36	40	1.0	710	7	20	1.0	40	7	10	1.0	250
	Morphine	53	2650	10.0	50,400	9	1330	20.0	11,700	12	310	90.0	5350
	Codeine	52	220	3.0	12,110	7	140	3.0	660	11	20	5.0	1210
Vitreous Humor	6-MAM	53	25	3.0	240	9	20	3.4	125	8	30	1.0	80
	6-Ac	27	2	Tr	20	6	4	1.0	20	2	10	1.0	10
	Morphine	53	90	10.0	890	9	90	10.0	260	9	120	11.0	300
	Codeine	52	10	1.0	140	9	10	3.0	20	8	10	200	30
Bile	6-MAM	8	10	2.0	40	6	10	10.0	40	3	10	1.0	160
	6-Ac	2	3	1.0	5	5	4	1.0	10	1	1	1.0	1
	Morphine	16	1590	130.0	41,100	6	4180	1510.0	9440	6	1060	40.0	403400
	Codeine	15	30	5.0	170	6	50	20.0	230	4	20	10	190

^&^ Analytes: 6-monoacetylmorphine (6-MAM), 6-acetylcodeine (6-AC); MOR: morphine; COD: codeine. ^#^ *N*: number of cases; * Tr: Trace concentration.

**Table 6:** Location of deaths and state of deceased body at autopsy in the 97 heroin-related fatalities cases in Jeddah, Saudi Arabia between 2008–2018.

		Location of Death								Putrefaction											
		Indoor				Outdoor				Non-Putrefied				Partial Putrefied				Heavy Putrefied			
Total Case Number		36				61				76				13				8			
	Analytes ^&^	*N ^#^*	Median	Minimum	Maximum	*N*	Median	Minimum	Maximum	*N*	Median	Minimum	Maximum	*N*	Median	Minimum	Maximum	*N*	Median	Minimum	Maximum
Blood with Sodium Fluoride	6-MAM	21	10	Tr *	420	29	10	1.0	150	46	10	Tr	420	2	10	1.0	10	2	10	3.0	20
	6-AC	9	4	Tr	30	11	2	Tr	10	20	3	Tr	30	0							
	MOR	33	320	20.0	4400	51	240	100.0	830	68	270	20.0	4400	10	320	121.0	725	6	370	200.0	720
	COD	33	20	3.0	140	51	20	3.0	110	68	20	3.0	140	10	20	3.0	60	6	20	4.0	70
Urine	6-MAM	30	310	10.0	18,900	44	360	1.0	4600	60	320	1.0	18,900	8	340	1.0	2520	6	540	130.0	1580
	6-AC	17	30	3.0	670	33	40	1.0	710	39	35	Tr	710	6	50	1.0	250	5	10	2.0	160
	MOR	30	1090	100.0	42,300	44	2030	10.0	50,400	60	1850	10.0	42,300	8	710	122.0	50,400	6	1140	520.0	4110
	COD	28	180	5.0	12,110	42	210	3.0	2100	56	200	3.0	12,110	8	50	3.0	1590	6	130	10.0	2100
Vitreous Humor	6-MAM	27	40	10.0	240	43	20	1.0	215	60	20	1.0	240	6	40	1.0	220	4	25	20.0	30
	6-AC	12	3	Tr	20	23	1	Tr	15	32	2	Tr	20	2	10	1.0	10	1	1	1.0	1
	MOR	27	100	10.0	490	44	80	10.0	890	60	70	10.0	890	6	220	11.0	550	5	140	40.0	300
	COD	26	20	3.0	70	43	10	1.0	140	58	10	1.0	140	6	15	2.0	30	5	10	3.0	10
Bile	6-MAM	9	10	2.0	40	8	10	1.0	160	12	10	1.0	160	3	10	2.0	40	2	10	2.0	10
	6-AC	3	1	1.0	3	5	5	1.0	10	6	2	Tr	10	1	4	4.0	4	1	5	5.0	5
	MOR	14	1075	40.0	7800	14	3520	120.0	41,100	21	1830	120.0	41,100	4	1520	40.0	4470	3	2150	490.0	40,400
	COD	12	20	5.0	120	13	70	10	230	19	30	5.0	230	3	40	13.0	40	3	70	10.0	190

^&^ Analytes: 6-monoacetylmorphine (6-MAM), 6-acetylcodeine (6-AC). ^#^ *N:* number of cases; * Tr: Trace concentration.

## Data Availability

The data underlying this article will be shared on reasonable request with the corresponding author.

## Supplementary Materials

The following supporting information can be downloaded at: https://www.mdpi.com/article/10.3390/toxics11030248/s1, Table S1: Summary of method validation parameters for the analysis of heroin biomarkers and their metabolites in blood, urine, vitreous humor, and bile; Table S2: Year distribution of 6-monoacetylmorphine, 6-acetylcodeine, morphine, and codeine in the 97 heroin-related deaths reported in Jeddah, Saudi Arabia, between 2008–2018; Table S3: Age distribution in the 97 heroin-related deaths examined in Jeddah, Saudi Arabia, between 2008–2018; Table S4: Concentrations of heroin biomarkers, morphine, and codeine in the 97 heroin related deaths in the current study according to post-mortem interval time group; Table S5: Comparison between heroin related metabolites concentrations in different bodily fluids according to the presence of injection marks at autopsy, presence of heroin materials in the scene and whether the last moment before death had been witnessed or they died alone in 97 heroin-related deaths in the current study; Table S6: Seasonal variation on the concentrations of heroin biomarkers, morphine, and codeine in the 97 heroin-related deaths in the current study; Table S7: Correlation between the heroin biomarkers, morphine, and codeine concentrations in the 97 heroin-related deaths in the current study; Figure S1: Variation of morphine concentration group in the blood with sodium fluoride (ng/mL) in the 97 heroin-related fatality cases in relation to postmortem interval time; Figure S2: Comparison between the median 6-monoacetylmorphine (ng/mL) in (a) blood with sodium fluoride and (b) vitreous humor samples in heroin-related deaths in the current study in relation to postmortem interval time; Figure S3: Distribution of heroin-related deaths in Jeddah, Saudi Arabia, between 2008–2018 (by month).
