# Supplementary material for: Heroin-Related Fatalities in Jeddah, Saudi Arabia, between 2008 and 2018
Source: Toxics. 2023 Mar 6;11(3):248. doi: 10.3390/toxics11030248 (PMC10099738; doi:10.3390/toxics11030248)
Supplement: Supplementary file 1 [file toxics-11-00248-s001.zip › toxics-2204660-supplementary.pdf]

Table S1. Summary **of** method validation parameters for the analysis of heroin biomarkers and their metabolites in blood, urine, vitreous humor and bile.

			Bodily fluid specimens															
			Blood with sodium fluoride				Urine				Vitreous humor				Bile			
	Analytes [&]		6-MAM	6-AC	MOR	COD	6-MAM	6-AC	MOR	COD	6-MAM	6-AC	MOR	COD	6-MAM	6-AC	MOR	COD
	LOD		0.4	0.2	0.2	0.2	0.2	0.2	0.3	0.3	0.2	0.3	0.2	0.2	0.3	0.3	0.4	0.3
	LLOQ	Mean (ng/g)	1	1	1	1	1	1	1	1	1	1	1	1	1	1	1	1
		%E [ҿ]	-2	4	1	2	5	-3	2	-5	-2	-6	-2	-2	-3	-2	-3	-1
		Within-run %CV**	9	5	5	6	6	9	3	11	4	10	5	10	13	8	9	12
		Between-run %CV	8	4	6	7	5	8	4	10	5	8	7	8	11	10	6	9
	LDR [Φ]	ng/mL	0.5-1000															
	R2 *		0.999	0.999	0.999	0.999	0.999	0.999	0.999	0.999	0.999	0.999	0.999	0.999	0.999	0.999	0.999	0.999
Accuracy and precision	Low control (5 ng/g)	Mean (ng/g)	5.0	4.9	4.9	4.9	4.9	5.0	5.0	4.8	4.9	4.9	4.8	4.8	5.0	5	5.2	5.2
		%E ҿ	-1	-3	-2	-2	-3	-1	-2	-4	-3	-2	-5	-3	1	3	5	3
		Within-run %CV**	14	11	9	7	11	13	6	10	11	12	10	8	10	14	13	11
		Between-run %CV	13	12	10	4	11	10	7	12	10	8	9	7	11	9	11	12
	Medium Control (100 ng/g)	Mean (ng/g)	98	98	102	97	98	101	99	98	99	102	99	99	103	102	102	95
		%E	-2	-2	2	-3	-2	1	-1	-2	-1	2	-1	-1	3	2	2	-5
		Within Run %CV	6	6	5	4	6	8	4	4	5	8	4	3	7	4	2	3
		Between Run %CV	9	8	8	2	7	4	6	6	2	8	8	4	9	7	7	5
	High control (800 ng/g)	Mean (ng/g)	784	788	810	791	779	786	789	788	793	788	778	812	784	777	793	796
		%E	-2	-1	1	-1	-3	-2	-1	-2	-1	-1	-3	2		-3	-1	-1

		Within Run %CV	4	2	4	2	3	2	2	2	2	3	2	4	-2	2	2	3
		Between Run %CV	3	3	4	2	1	2	2	3	3	4	2	1	2	2	3	1
Dilution	100 ng/g Ψ	Mean (ng/mL)	9.9	9.5	9.6	9.9	9.8	10	9.7	9.5	9.7	10	9.6	9.7	10.2	10.1	10.2	10.1
		%E	-1	-5	-4	-1	-2	-1	-3	-5	-3	1	-4	-3	2	1	2	1
		Within-run %CV	7	9	3	5	10	6	6	4	12	13	5	6	8	5	5	6
		Between-run %CV	6	8	4	4	9	5	6	4	8	7	4	5	8	6	7	5
	7500 ng/g [¥]	Mean (ng/mL)	76	79	78	74	76	78	76	77	79	79	77	74	78	76	78	79
		%E	1	5	4	-1	1	4	1	2	-1	6	2	-1	-2	2	4	6
		Within-run %CV	5	2	6	6	7	5	7	8	2	5	4	7	2	3	2	3
		Between-run %CV	4	3	5	9	5	4	4	7	3	8	3	7	3	1	3	1
Matrix effects	5 ng/mL	Mean (%)	3	-2	18	8	9	-3	-12	5	-4	8	14	3	7	13	14	13
		%CV	5	3	3	5	3	2	4	1	5	4	1	1	6	3	5	3
Recovery		Mean (%)	92	95	86	92	90	89	99	99	86	89	98	99	91	87	95	92
		%CV	4	1	4	7	2	2	5	8	7	10	6	2	6	4	4	2
Matrix effects (%)	100 ng/mL	Mean (%)	7	10	19	2	1	8	-13	-1	-7	6	7	2	4	5	20	2
		%CV	5	6	7	5	6	4	7	1	4	3	6	4	5	2	4	5
Recovery (%)		Mean (%)	98	96	98	94	97	98	99	93	94	95	95	98	95	97	97	97
		%CV	7	7	6	7	6	7	6	8	5	8	5	3	6	5	5	7
Matrix effects (%)	800 ng/mL	Mean (%)	7	10	19	2	1	8	-13	-1	-7	6	7	2	4	5	20	2
		%CV	5	6	7	5	6	4	7	1	4	3	6	4	5	2	4	5
Recovery (%)		Mean (%)	98	96	98	94	97	98	99	93	94	95	95	98	95	97	97	97

		%CV	7	7	6	7	6	7	6	8	5	8	5	3	6	5	5	7
Autosampler Stability (5 ng/mL)	24 h	Mean (%)	99	98	97	98	100	100	99	100	97	95	98	99	96	99	101	101
		%CV	10	5	9	7	4	6	7	3	8	4	11	5	6	11	5	12
	48 h	Mean (%)	99	97	98	100	99	99	97	102	98	98	98	99	98	97	104	103
		%CV	8	3	7	6	5	4	6	2	3	5	7	6	9	3	4	11
	One week	Mean (%)	98	94	101	101	99	98	98	101	99	96	100	100	98	96	102	104
		%CV	6	3	5	11	9	7	6	5	11	8	9	12	10	12	8	3
Autosampler Stability (100 ng/mL)	24 h	Mean (%)	100	98	100	103	98	98	100	100	100	99	99	100	99	98	100	102
		%CV	8	4	7	3	5	6	5	4	3	2	4	5	2	4	3	3
	48 h	Mean (%)	98	96	102	105	97	97	101	103	99	98	100	101	99	98	101	105
		%CV	4	3	5	6	5	3	6	4	2	5	3	2	4	3	7	2
	One week	Mean (%)	97	96	103	104	96	97	102	104	98	97	101	101	98	97	101	101
		%CV	4	3	7	4	3	2	4	6	4	6	3	2	4	2	5	1
Autosampler Stability (800 ng/mL)	24 h	Mean (%)	100	100	100	101	100	100	100	100	100	100	100	100	100	100	100	100
		%CV	2	3	1	1	2	1	2	2	3	2	1	1	2	1	1	3
	48 h	Mean (%)	99	99	101	100	99	100	99	100	100	100	100	101	99	99	100	101
		%CV	2	1	1	2	2	2	2	1	4	1	2	1	3	1	2	2
	One week	Mean (%)	99	98	100	100	99	99	99	100	100	100	100	101	99	99	100	101
		%CV	1	3	1	2	2	3	2	1	4	1	2	1	3	1	2	2

[&] Analytes: 6-monoacetylmorphine (6-MAM), 6-acetylcodeine (6-AC); MOR: morphine; COD: codeine ; * R2: Coefficient of determination; Φ LDR: Linear dynamic range; **CV: coefficient of variation; ҿ %E: Relative error.Ψ:Dilution factor: 1:10 times; ¥ Dilution factor: 1:100 times; # %: percentage comparing to targeted concentration.

Table S2. Year distribution of 6-monoacetylmorphine, 6-acetylcodeine, morphine, and codeine in the 97 heroin-related deaths cases reported in Jeddah, Saudi Arabia between 2008–2018.

			Blood with sodium fluoride				Urine				Vitreous humor				Bile			
Year	Total $N^{\#}$		6-MAM	6-AC	Morphine	Codeine	6-MAM	6-AC	Morphine	Codeine	6-MAM	6-AC	Morphine	Codeine	6-MAM	6-AC	Morphine	Codeine
2008	4	*N*	4	0	4	4	3	3	3	3	2	1	2	2	1	0	1	1
		Median	7	.	141	12	87	12	213	279	37	2	121	6	1	.	121	7
		Minimum	1.3	.	104.0	7.0	42.0	1.2	186.0	71.0	1.3	1.8	120.0	5.0	1.3	.	121.0	7.0
		Maximum	14	.	219	20	426	13	9279	602	72	2	121	7	1	.	121	7
2009	10	*N*	2	0	8	8	6	3	6	6	8	5	9	9	2	1	2	2
		Median	2	.	215	9	26	3	1515	103	13	1	123	13	12	1	4575	40
		Minimum	1.2	.	23.0	4.0	1.0	2.0	122.0	5.0	1.0	Tr*	11.0	2.0	12.0	1.0	1350.0	12.0
		Maximum	2	.	314	56	670	69	11700	660	33	8	270	72	12	1	7800	67
2010	9	*N*	4	1	5	5	7	3	7	6	7	2	7	5	0	0	2	2
		Median	11	2	290	33	471	320	6338	417	14	12	63	12	.	.	41091	165
		Minimum	4.0	1.6	75.0	3.0	228.0	87.0	242.0	100.0	6.0	1.0	17.0	3.0	.	.	41081.0	165.0
		Maximum	23	2	420	65	4557	709	20424	972	125	23	254	39	.	.	41100	165
2011	10	*N*	8	3	9	9	5	1	5	5	6	3	6	6	0	0	0	0
		Median	21	6	290	45	130	56	3836	300	116	4	77	24	.	.	.	.
		Minimum	1.0	5.0	121.0	11.0	5.0	56.0	14.0	8.0	25.0	3.0	8.0	8.0	.	.	.	.
		Maximum	153	11	827	76	2778	56	6933	904	196	8	891	143	.	.	.	.
2012	7	*N*	5	3	7	7	6	5	6	6	4	2	4	4	0	0	1	1
		Median	21	4	470	42	401	35	3312	252	49	1	117	21	.	.	133	14
		Minimum	6.0	1.0	278.0	5.0	14.0	11.0	102.0	23.0	18.0	Tr	44.0	11.0	.	.	133.0	14.0
		Maximum	251	19	683	76	2778	55	7498	1303	140	1	244	76	.	.	133	14
2013	6	*N*	3	1	5	5	6	4	6	6	5	4	5	5	2	0	2	2
		Median	8	1	340	28	345	13	1826	124	25	1	160	17	26	.	1405	31
		Minimum	5.0	1.3	105.0	4.0	65.0	3.0	720.0	6.0	10.0	.5	37.0	9.0	14.0	.	459.0	24.0
		Maximum	10	1	439	79	2708	34	15953	1130	45	2	255	25	38	.	2350	37
2014	7	*N*	5	2	7	7	6	3	6	6	6	4	6	6	1	0	3	3

		Median	10	22	369	41	107	41	1072	87	94	5	110	15	10	.	1264	23
		Minimum	3.0	14.0	108.0	18.0	10.0	5.0	96.0	5.0	21.0	.6	39.0	9.0	10.0	.	494.0	5.0
		Maximum	416	29	4387	140	2391	135	50401	1593	243	13	545	124	10	.	9808	79
2015	8	*N*	4	1	8	8	6	4	6	6	3	1	3	3	1	1	1	1
		Median	10	4	354	32	99	21	377	34	64	3	148	18	9	1	412	37
		Minimum	6.0	4.0	190.0	18.0	1.3	1.0	184.0	12.0	26.0	3.0	17.0	4.0	9.0	.9	412.0	37.0
		Maximum	266	4	1927	63	18876	670	42264	12110	70	3	390	27	9	1	412	37
2016	12	*N*	4	2	11	11	7	4	7	7	10	3	10	10	1	0	3	2
		Median	15	2	250	18	1315	341	848	54	28	2	71	22	160	.	5980	98
		Minimum	10.0	Tr	119.0	7.0	48.0	39.0	86.0	7.0	14.0	2.0	17.0	3.0	160.0	.	1715.0	6.5
		Maximum	104	5	715	67	3390	641	12247	903	212	13	201	31	160	.	40383	189
2017	9	*N*	4	1	8	8	7	6	7	7	7	4	7	7	4	2	5	4
		Median	6	1	222	10	520	96	1325	102	22	8	42	9	8	5	1509	29
		Minimum	.7	1.0	112.0	3.0	91.0	8.0	524.0	31.0	3.4	.4	13.0	3.0	2.0	5.0	40.0	13.0
		Maximum	23	1	514	54	1584	201	4114	555	65	15	260	14	14	5	2577	66
2018	15	*N*	7	6	12	12	15	14	15	12	12	6	12	12	5	4	8	7
		Median	12	2	233	16	358	19	625	195	18	3	92	9	13	4	3366	36
		Minimum	.3	.2	122.0	3.0	1.0	1.0	23.0	3.0	3.0	Tr	8.0	1.4	2.0	1.3	300.0	6.0
		Maximum	46	9	670	114	3734	519	17800	1214	96	6	487	67	41	8	9438	227

[&] Analytes: 6-monoacetylmorphine (6-MAM), 6-acetylcodeine (6-AC); MOR: morphine; COD: codeine
[#]*N*: number of cases; *****Tr: Trace concentration

Table S3. Age distribution in the 97 heroin-related deaths examined in Jeddah, Saudi Arabia between 2008–2018.

			Blood with sodium fluoride				Urine				Vitreous humor				Bile			
Age (year)	Total *N*#		6-MAM	6-AC	Morphine	Codeine	6-MAM	6-AC	Morphine	Codeine	6-MAM	6-AC	Morphine	Codeine	6-MAM	6-AC	Morphine	Codeine
10-20	4	*N*	1	0	4	4	4	3	4	3	2	1	2	2	3	2	3	3
		Median	10	.	250	25	221	14	1281	12	31	1	151	17	14	4	2350	37
		Minimum	10.0	.	150.0	10.0	63.0	1.0	376.7	12.0	17.0	1.0	47.0	12.0	9.0	1.0	412.0	24.0
		Maximum	10	.	361	30	482	34	2301	141	45	1	255	21	41	8	9438	227
21-30	32	*N*	15	7	27	27	28	21	28	27	25	13	26	26	7	4	12	10
		Median	7	2	250	19	335	35	1282	211	18	2	74	17	13	4	4518	50
		Minimum	.3	Tr*	105.0	3.0	10.0	1.0	23.0	3.0	3.0	Tr	8.0	3.0	2.0	1.3	885.0	6.0
		Maximum	266	9	1927	114	18876	709	42264	12110	91	13	390	124	160	5	41100	165
31-40	26	*N*	17	5	23	23	19	12	19	19	22	11	22	21	3	1	6	5
		Median	10	4	255	18	340	56	1742	178	26	2	66	13	13	5	1144	37
		Minimum	1.3	Tr	75.0	3.0	5.0	1.2	14.0	6.0	1.3	Tr	8.0	3.0	1.3	5.0	40.0	7.0
		Maximum	153	11	715	76	1748	589	11108	1303	196	23	487	67	38	5	40383	189
41-50	20	*N*	11	5	18	18	13	7	13	11	13	5	13	12	3	0	5	5
		Median	24	2	369	23	701	89	687	200	32	1	109	11	2	.	494	13
		Minimum	1.0	Tr	104.0	4.0	1.0	2.0	45.0	3.0	8.0	Tr	37.0	1.4	2.0	.	300.0	5.0
		Maximum	160	29	869	67	3734	340	17800	605	243	13	302	31	10	.	2577	33
51-70	15	*N*	6	3	12	12	10	7	10	10	8	5	8	8	1	1	2	2
		Median	21	14	302	34	136	33	2245	187	52	2	137	15	12	1	742	41
		Minimum	1.0	5.0	23.0	3.0	1.0	2.0	122.0	5.0	1.0	Tr	11.0	2.0	12.0	1.0	133.0	14.0
		Maximum	416	19	4387	140	2521	249	50401	2100	215	8	891	143	12	1	1350	67

& Analytes: 6-monoacetylmorphine (6-MAM), 6-acetylcodeine (6-AC); MOR: morphine; COD: codeine

#N: number of cases; *Tr: Trace concentration

Table S4. Concentrations of heroin biomarkers, morphine, and codeine in the 97 heroin related deaths in the current study according to post-mortem interval time group.

			Blood with sodium fluoride				Urine				Vitreous humor				Bile			
Post-mortem Intervals (h)	Total *N*		6-MAM	6-AC	MOR	COD	6-MAM	6-AC	MOR	COD	6-MAM	6-AC	MOR	COD	6-MAM	6-AC	MOR	COD
<24	49	*N*[#]	32	20	44	44	37	26	37	33	44	24	44	43	7	4	14	12
		Median	16	3	255	21	324	35	1963	207	22	3	70	14	12	4	3366	35
		Minimum	Tr*	Tr	23.0	3.0	1.0	1.0	14.0	3.0	3.0	Tr	8.0	1.4	2.0	1.3	133.0	6.0
		Maximum	416	29	4387	140	18876	709	42264	12110	243	23	891	143	41	8	41100	227
25-48 h	18	*N*	7	0	16	16	16	12	16	16	14	7	14	13	6	2	6	6
		Median	6		314	22	403	35	2200	154	19	1	121	11	14	3	1430	31
		Minimum	1.3		105.0	4.0	1.0	1.2	122.0	5.0	1.0	Tr	11.0	2.0	1.3	1.0	121.0	6.5
		Maximum	10		725	114	2391	134	50401	1593	215	8	545	76	160	5	5980	67
49-72 h	10	*N*	5	0	9	9	9	3	9	9	6	2	6	6	0	0	1	1
		Median	5		277	24	67	135	584	38	37	8	93	22			9808	79
		Minimum	2.0		108.0	7.0	1.3	12.7	102.0	12.0	14.0	2.0	17.0	3.0			9808.0	79.0
		Maximum	21		429	76	3390	641	12247	904	91	13	254	124			9808	79
73-120	8	*N*	3	0	5	5	3	3	3	3	2	0	2	2	1	0	1	1
		Median	1		370	45	851	56	5351	532	50		266	15	2		2577	13
		Minimum	1.0		121.0	11.0	138.0	3.0	931.0	39.0	30.0		142.0	11.0	2.0		2577.0	13.0
		Maximum	12		514	67	2521	249	6933	1214	70		390	18	2		2577	13
>120	12	*N*	3	0	10	10	9	6	9	9	4	2	5	5	3	2	6	5
		Median	10		346	23	520	51	704	72	25	8	160	8	9	3	876	37
		Minimum	3.0		190.0	4.0	58.0	1.0	376.7	12.0	22.0	1.0	42.0	3.0	2.0	.9	40.0	5.0
		Maximum	17		869	46	1584	201	4114	2100	41	14	302	14	13	5	40383	189

[&] Analytes: 6-monoacetylmorphine (6-MAM), 6-acetylcodeine (6-AC); MOR: morphine; COD: codeine
[#]*N*: number of cases; *Tr: Trace concentration

Table S5. Comparison between heroin related metabolites concentrations in different bodily fluids ac-cording to the presence of injection marks at autopsy, presence of heroin materials in the scene and whether the last moment before death had been witnessed or they died alone in 97 hero-in-related deaths in the current study.

				Blood with sodium fluoride				Urine				Vitreous humor				Bile			
		Total N#		6-MAM	6-AC	MOR	COD	6-MAM	6-AC	MOR	COD	6-MAM	6-AC	MOR	COD	6-MAM	6-AC	MOR	COD
Injection Marks	No	30	N	14	6	24	24	27	18	27	26	20	11	21	19	11	6	15	13
			Median	7	1	215	13	283	14	704	107	23	2	121	9	13	4	2151	37
			Minimum	Tr*	Tr	75.0	3.0	1.3	.7	45.0	3.0	1.3	.2	13.0	1.4	1.3	.9	40.0	6.0
			Maximum	23	3	715	79	4557	709	20424	1130	125	23	302	29	160	5	41081	189
	Yes	67	N	36	14	60	60	47	32	47	44	50	24	50	50	6	2	13	12
			Median	13	5	290	28	471	42	2185	208	26	2	71	14	11	4	1829	27
			Minimum	1.0	Tr	23.0	3.0	1.0	1.0	14.0	5.0	1.0	Tr	8.0	2.0	2.0	1.0	133.0	5.0
			Maximum	416	29	4387	140	18876	670	50401	12110	243	15	891	143	41	8	41100	227
Heroin powder presence	No	80	N	44	19	72	72	61	42	61	57	58	29	59	58	15	8	25	23
			Median	10	3	278	21	324	36	1238	166	26	2	75	13	12	4	1715	37
			Minimum	Tr	Tr	23.0	3.0	1.0	1.0	14.0	3.0	1.0	Tr	8.0	1.4	1.3	.5	40.0	5.0
			Maximum	416	29	4387	140	4557	709	20424	2100	243	23	891	143	160	8	41100	227
	Yes	17	N	6	1	12	12	13	8	13	13	12	6	12	11	2	0	3	2
			Median	12	4	337	31	378	38	2788	220	18	1	155	17	13		2831	18
			Minimum	1.2	4.0	104.0	4.0	14.0	3.0	102.0	23.0	10.0	.3	8.0	5.0	12.0		2350.0	12.0
			Maximum	266	4	1927	76	18876	670	50401	12110	215	3	545	27	14		7800	24
Final moments before death	Witnesses	34	N	24	10	32	32	26	19	26	25	27	16	28	28	10	5	13	10
			Median	7	4	270	21	335	35	1644	143	25	3	72	14	13	1	1715	31
			Minimum	Tr	Tr	105.0	4.0	5.0	1.2	14.0	7.0	1.3	Tr	8.0	3.0	1.3	.5	40.0	6.0
			Maximum	416	14	4387	140	18876	709	42264	12110	196	15	891	143	160	5	7800	120
	No witnesses	63	N	26	10	52	52	48	31	48	45	43	19	43	41	7	3	15	15
			Median	12	2	302	22	332	39	1404	208	24	1	92	13	10	5	2151	36
			Minimum	1.0	Tr	23.0	3.0	1.0	1.0	45.0	3.0	1.0	.2	11.0	1.4	2.0	4.0	133.0	5.0
			Maximum	251	29	869	114	3734	641	50401	2100	243	23	545	124	41	8	41100	227

& Analytes: 6-monoacetylmorphine (6-MAM), 6-acetylcodeine (6-AC); MOR: morphine; COD: codeine
N: number of cases; Tr: Trace concentration

Table S6. Seasonal variation on the concentrations of heroin biomarkers, morphine, and codeine in the 97 heroin-related deaths in the current study.

Months	Total $N^{\#}$	Analytes [&]	6-MAM	6-AC	MOR	COD	6-MAM	6-AC	MOR	COD	6-MAM	6-AC	MOR	COD	6-MAM	6-AC	MOR	COD
January	10	*N*	5	2	9	9	10	8	10	9	7	2	7	7	3	2	3	3
		Median	6	5	365	24	559	36	2316	284	22	3	68	14	9	5	2151	36
		Minimum	2.0	1.0	122.0	3.0	18.0	1.0	23.0	3.0	5.0	Tr*	10.0	3.0	2.0	4.0	488.0	13.0
		Maximum	41	9	683	114	2778	162	5844	904	140	5	244	76	13	5	4468	66
February	10	*N*	5	3	8	8	8	6	8	8	8	5	8	8	4	3	6	6
		Median	4	2	300	22	235	19	847	116	22	3	106	13	14	3	4235	87
		Minimum	Tr	Tr	75.0	3.0	14.0	2.0	102.0	12.0	3.4	.4	13.0	3.0	2.0	1.3	885.0	6.0
		Maximum	21	3	429	42	2582	87	6338	2100	125	23	270	27	19	5	41100	165
March	10	*N*	6	3	9	9	5	3	5	5	8	5	8	8	2	0	4	3
		Median	17	11	271	28	378	34	1457	141	36	1	98	22	87	.	2033	7
		Minimum	6.0	1.6	119.0	4.0	58.0	12.0	86.0	7.0	19.0	Tr	8.0	4.0	14.0	.	1264.0	5.0
		Maximum	416	14	4387	140	4557	709	20424	972	196	8	255	30	160	.	5980	24
April	11	*N*	6	4	9	9	7	5	7	7	8	5	8	8	2	0	3	3
		Median	79	5	449	24	42	2	244	71	14	3	42	12	6	.	494	23
		Minimum	1.3	3.5	121.0	7.0	1.0	1.0	96.0	5.0	1.0	1.0	11.0	2.0	1.3	.	121.0	7.0
		Maximum	266	29	1927	63	18876	670	42264	12110	243	8	167	72	10	.	40383	189
May	8	*N*	4	1	6	6	5	2	5	5	7	3	7	7	1	1	3	2
		Median	6	2	232	16	48	39	2098	166	30	1	92	8	12	1	1350	50
		Minimum	1.0	2.0	121.0	4.0	1.0	8.0	76.0	5.0	13.0	1.0	8.0	3.0	12.0	1.0	300.0	33.0
		Maximum	24	2	370	67	822	69	11700	660	89	8	150	31	12	1	2831	67
June	8	*N*	4	1	8	8	6	6	6	6	6	4	6	6	0	0	0	0
		Median	4	0	255	23	124	10	1574	123	9	1	126	12				
		Minimum	1.0	Tr	23.0	3.0	24.0	2.0	45.0	3.0	6.0	Tr	17.0	1.4				
		Maximum	12	0	725	54	2391	320	50401	1593	215	1	545	21				
July	12	*N*	6	2	9	9	9	5	9	8	7	3	8	6	2	0	3	3
		Median	14	10	190	20	324	33	3195	257	14	2	62	16	7	.	2577	13
		Minimum	1.2	Tr	104.0	5.0	20.0	1.0	242.0	12.0	10.0	1.0	32.0	9.0	2.0	.	133.0	12.0
		Maximum	251	19	313	40	1748	589	11108	634	79	15	184	18	12	.	7800	14
August	7	*N*	2	2	5	5	7	4	7	7	3	3	3	3	1	1	2	1
		Median	56	3	319	30	340	147	1337	200	41	13	201	9	9	1	226	37
		Minimum	8.0	1.0	240.0	13.0	1.3	56.0	184.0	12.0	6.0	1.0	38.0	8.0	9.0	1.0	40.0	37.0
		Maximum	104	5	478	35	2521	249	5351	1214	212	14	260	21	9	1	412	37
September	7	*N*	2	0	7	7	6	5	6	6	5	1	6	6	0	0	1	1
		Median	6		250	11	939	89	1030	201	25	2	68	13			1829	31

		Minimum	4.3		121.0	8.0	5.0	39.0	14.0	8.0	14.0	2.0	17.0	5.0			1829.0	31.0
		Maximum	7		360	54	3390	641	12247	903	71	2	487	67			1829	31
October	3	*N*	2	1	3	3	3	2	3	2	2	1	1	1	0	0	0	0
		Median	27	1	474	76	2708	192	15953	675	61	2	190	9				
		Minimum	8.0	1.3	340.0	41.0	238.0	43.0	2788.0	220.0	25.0	2.3	190.0	9.0				
		Maximum	46	1	647	79	3734	340	17800	1130	96	2	190	9				
November	4	*N*	2	0	4	4	3	1	3	3	3	0	3	3	1	0	1	1
		Median	27		453	21	1434	13	848	51	70		102	18	38	.	459	37
		Minimum	12.0		167.0	4.0	65.0	13.0	720.0	6.0	26.0		68.0	11.0	38.0	.	459.0	37.0
		Maximum	41		683	47	2778	13	3836	300	140		390	27	38	.	459	37
December	7	*N*	6	1	7	7	5	3	5	4	6	3	6	6	1	1	2	2
		Median	8	5	160	18	426	14	1023	78	53	4	187	32	41	8	9623	153
		Minimum	3.0	5.0	105.0	10.0	10.0	12.0	104.0	12.0	16.0	1.8	37.0	5.0	41.0	8.0	9438.0	79.0
		Maximum	35	5	827	76	943	39	9279	602	133	13	891	143	41	8	9808	227

[&] Analytes: 6-monoacetylmorphine (6-MAM), 6-acetylcodeine (6-AC); MOR: morphine; COD: codeine

[#]*N*: number of cases; *Tr: Trace concentration

Table S7. Correlation between the heroin biomarkers, morphine, and codeine concentrations in the 97 heroin-related death in the current study.

			Blood with Sodium Fluoride				Urine				Vitreous Humor				Bile			
	Analytes &		6-MAM	6-AC	MOR	COD	6-MAM	6-AC	MOR	COD	6-MAM	6-AC	MOR	COD	6-MAM	6-AC	MOR	COD
Blood with Sodium Fluoride	6-MAM	Rs	1.000	.699**	.510**	.336*	.333*	.435*	.204	.131	.532**	-.016	-.064	.243	-.072	.211	-.389	-.095
		Sig. (2-tailed)	.	<.001	<.001	.017	.041	.034	.220	.454	<.001	.940	.697	.141	.854	.789	.169	.747
		Number of cases	50	20	50	50	38	24	38	35	40	24	39	38	9	4	14	14
	6-AC	Rs	.699**	1.000	.179	.222	-.150	.182	-.085	.052	.539*	.537*	-.107	.399	.000	-1.000	-.600	-.143
		Sig. (2-tailed)	<.001	.	.450	.348	.579	.553	.753	.854	.021	.026	.671	.101	1.000	.	.208	.787
		Number of cases	20	20	20	20	16	13	16	15	18	17	18	18	4	2	6	6
	MOR	Rs	.510**	.179	1.000	.631**	.285*	.213	.269*	.262*	.428**	-.243	.295*	.038	-.076	-.643	-.315	-.183
		Sig. (2-tailed)	<.001	.450	.	<.001	.023	.166	.031	.043	<.001	.172	.018	.768	.779	.086	.133	.415
		Number of cases	50	20	84	84	64	44	64	60	63	33	64	63	16	8	24	22
	COD	Rs	.336*	.222	.631**	1.000	.274*	.383*	.301*	.387**	.426**	.000	.190	.258*	.218	-.217	.164	.001
		Sig. (2-tailed)	.017	.348	<.001	.	.028	.010	.016	.002	<.001	1.000	.132	.041	.416	.606	.444	.997
		Number of cases	50	20	84	84	64	44	64	60	63	33	64	63	16	8	24	22
Urine	6-MAM	Rs	.333*	-.150	.285*	.274*	1.000	.782**	.651**	.591**	.398**	.164	.180	.001	.359	.214	.138	.377
		Sig. (2-tailed)	.041	.579	.023	.028	.	<.001	<.001	<.001	.002	.394	.185	.993	.189	.610	.519	.092
		Number of cases	38	16	64	64	74	50	74	70	56	29	56	54	15	8	24	21
	6-AC	Rs	.435*	.182	.213	.383*	.782**	1.000	.488**	.446**	.401*	.448*	-.004	.020	.087	.000	-.141	.158
		Sig. (2-tailed)	.034	.553	.166	.010	<.001	.	<.001	.002	.014	.032	.980	.907	.800	1.000	.602	.589
		Number of cases	24	13	44	44	50	50	50	47	37	23	37	37	11	7	16	14
	MOR	Rs	.204	-.085	.269*	.301*	.651**	.488**	1.000	.838**	.171	-.156	.150	.033	.461	.452	.317	.128
		Sig. (2-tailed)	.220	.753	.031	.016	<.001	<.001	.	<.001	.209	.420	.271	.810	.084	.260	.132	.580
		Number of cases	38	16	64	64	74	50	74	70	56	29	56	54	15	8	24	21
	COD	Rs	.131	.052	.262*	.387**	.591**	.446**	.838**	1.000	.118	-.063	.076	-.044	.252	.036	.231	.023
		Sig. (2-tailed)	.454	.854	.043	.002	<.001	.002	<.001	.	.404	.749	.587	.759	.384	.939	.290	.922
		Number of cases	35	15	60	60	70	47	70	70	52	28	53	52	14	7	23	20
Vitreous Humor	6-MAM	Rs	.532**	.539*	.428**	.426**	.398**	.401*	.171	.118	1.000	.509**	.346**	.371**	.343	-.714	-.071	-.058
		Sig. (2-tailed)	<.001	.021	<.001	<.001	.002	.014	.209	.404	.	.002	.004	.002	.231	.071	.760	.820
		Number of cases	40	18	63	63	56	37	56	52	70	35	69	67	14	7	21	18
	6-AC	Rs	-.016	.537*	-.243	.000	.164	.448*	-.156	-.063	.509**	1.000	-.116	.136	-.265	-1.000**	-.084	.434
		Sig. (2-tailed)	.940	.026	.172	1.000	.394	.032	.420	.749	.002	.	.505	.435	.612	.	.830	.283
		Number of cases	24	17	33	33	29	23	29	28	35	35	35	35	6	3	9	8
	MOR	Rs	-.064	-.107	.295*	.190	.180	-.004	.150	.076	.346**	-.116	1.000	.369**	.075	-.714	.155	.170
		Sig. (2-tailed)	.697	.671	.018	.132	.185	.980	.271	.587	.004	.505	.	.002	.799	.071	.502	.499
		Number of cases	39	18	64	64	56	37	56	53	69	35	71	69	14	7	21	18
	COD	Rs	.243	.399	.038	.258*	.001	.020	.033	-.044	.371**	.136	.369**	1.000	.351	-.346	.288	-.080

		Sig. (2-tailed)	.141	.101	.768	.041	.993	.907	.810	.759	.002	.435	.002	.	.218	.448	.205	.753
		Number of cases	38	18	63	63	54	37	54	52	67	35	69	69	14	7	21	18
Bile	6-MAM	Rs	-.072	.000	-.076	.218	.359	.087	.461	.252	.343	-.265	.075	.351	1.000	.566	.509*	.495*
		Sig. (2-tailed)	.854	1.000	.779	.416	.189	.800	.084	.384	.231	.612	.799	.218	.	.143	.037	.043
		Number of cases	9	4	16	16	15	11	15	14	14	6	14	14	17	8	17	17
	6-AC	Rs	.211	-1.000**	-.643	-.217	.214	.000	.452	.036	-.714	-1.000**	-.714	-.346	.566	1.000	.571	.119
		Sig. (2-tailed)	.789	.	.086	.606	.610	1.000	.260	.939	.071	.	.071	.448	.143	.	.139	.779
		Number of cases	4	2	8	8	8	7	8	7	7	3	7	7	8	8	8	8
	MOR	Rs	-.389	-.600	-.315	.164	.138	-.141	.317	.231	-.071	-.084	.155	.288	.509*	.571	1.000	.537**
		Sig. (2-tailed)	.169	.208	.133	.444	.519	.602	.132	.290	.760	.830	.502	.205	.037	.139	.	.006
		Number of cases	14	6	24	24	24	16	24	23	21	9	21	21	17	8	28	25
	COD	Rs	-.095	-.143	-.183	.001	.377	.158	.128	.023	-.058	.434	.170	-.080	.495*	.119	.537**	1.000
		Sig. (2-tailed)	.747	.787	.415	.997	.092	.589	.580	.922	.820	.283	.499	.753	.043	.779	.006	.
		Number of cases	14	6	22	22	21	14	21	20	18	8	18	18	17	8	25	25

& Analytes: 6-monoacetylmorphine (6-MAM), 6-acetylcodeine (6-AC); MOR: morphine; COD; # Rs. Correlation Coefficient.; **. Correlation is significant at the 0.01 level (2-tailed); *. Correlation is significant at the 0.05 level (2-tailed).

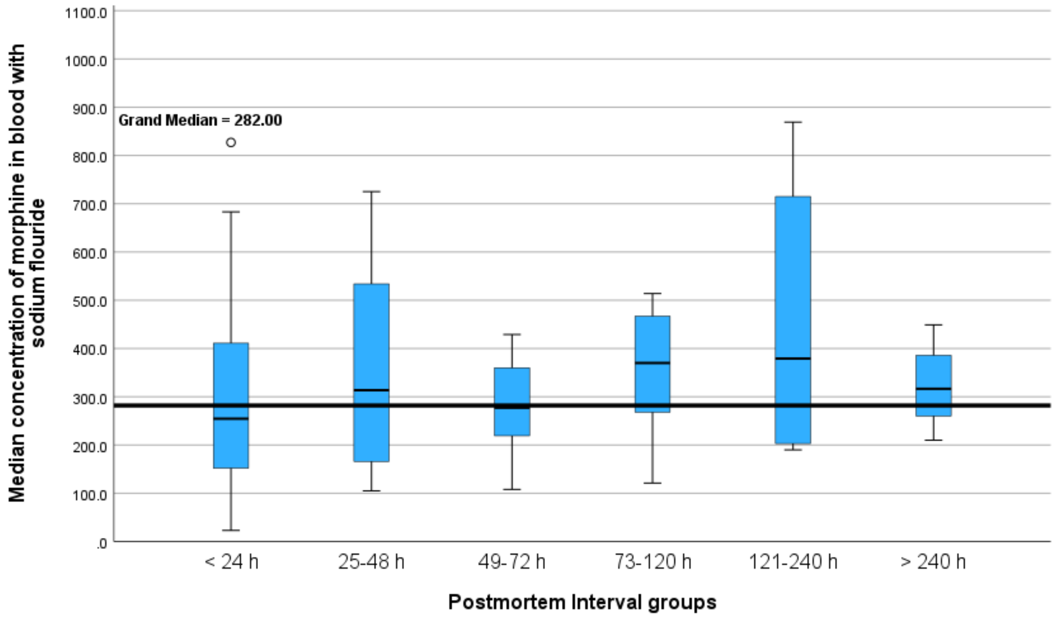

Figure S1. Variation of morphine concentration group in the blood with sodium fluoride (ng/mL) in the 97 heroin-related fatality cases in relation to postmortem interval time

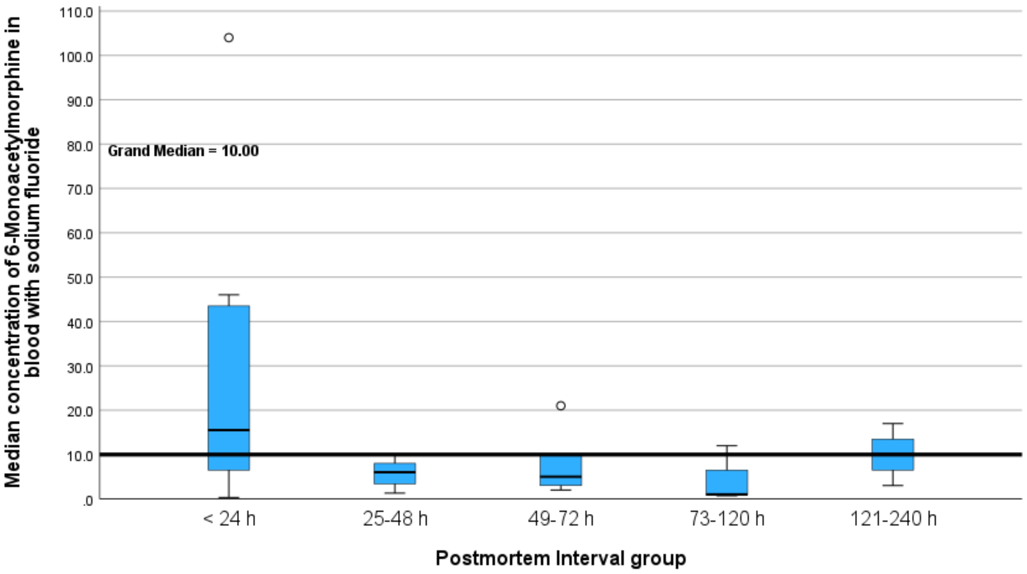

(a)

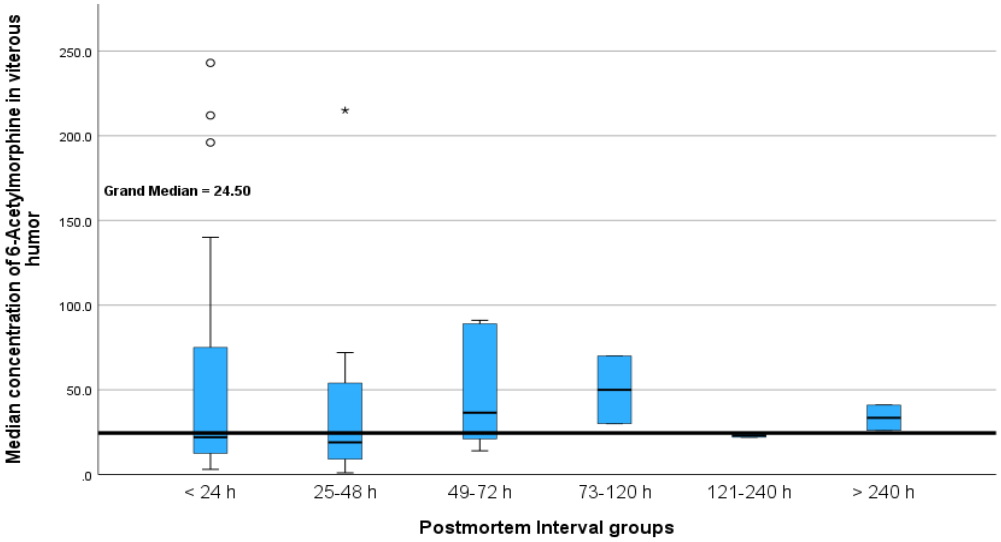

(b)

Figure S2. Comparison between the median 6-monoacetylmorphine (ng/mL) in (a) blood with sodium fluoride and (b) vitreous humor samples in heroin-related deaths in the current study in relation to postmortem interval time.

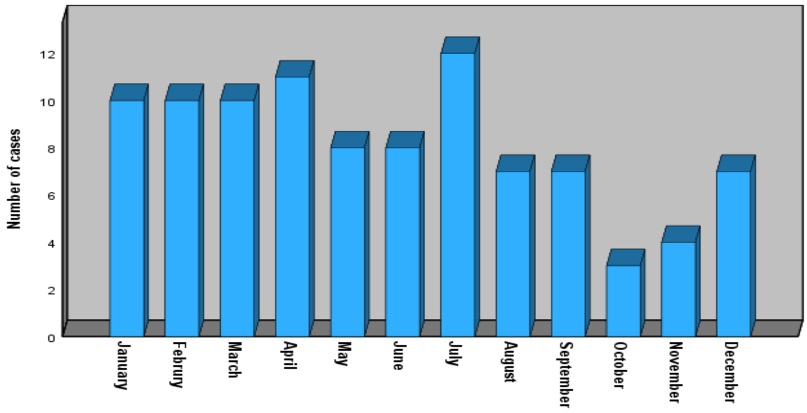

Figure S3 Distribution of heroin-related deaths in Jeddah, Saudi Arabia between 2008–2018 (by month).
